# Non‐Thermal Plasma as a Unique Delivery System of Short‐Lived Reactive Oxygen and Nitrogen Species for Immunogenic Cell Death in Melanoma Cells

**DOI:** 10.1002/advs.201802062

**Published:** 2019-01-28

**Authors:** Abraham Lin, Yury Gorbanev, Joey De Backer, Jinthe Van Loenhout, Wilma Van Boxem, Filip Lemière, Paul Cos, Sylvia Dewilde, Evelien Smits, Annemie Bogaerts

**Affiliations:** ^1^ Plasma, Laser Ablation, and Surface Modeling—Antwerp (PLASMANT) University of Antwerp Universiteitsplein 1 2610 Antwerpen‐Wilrijk Belgium; ^2^ Center for Oncological Research (CORE) University of Antwerp Universiteitsplein 1 2610 Antwerpen‐Wilrijk Belgium; ^3^ Department of Biomedical Sciences University of Antwerp Universiteitsplein 1 2610 Antwerpen‐Wilrijk Belgium; ^4^ Biomolecular and Analytical Mass Spectrometry (BAMS) Group Department of Chemistry & Centre for Proteomics University of Antwerp Groenenborgerlaan 171 2020 Antwerpen Belgium; ^5^ Department of Pharmaceutical Sciences University of Antwerp Universiteitsplein 1 2610 Antwerpen‐Wilrijk Belgium

**Keywords:** cancer, cancer immunotherapy, immunogenic cell death, melanoma, non‐thermal plasma, reactive oxygen species

## Abstract

Breakthroughs in cancer immunotherapies have demonstrated considerable success, though not without limitations. Non‐thermal plasma (NTP) for cancer therapy has been emerging as a potential adjuvant treatment via induction of immunogenic cell death (ICD). Cancer cells undergoing ICD stimulate a patient's immune system to mount an anticancer response. While promising, the underlying mechanisms of NTP‐induced ICD must be closely examined. Here, the interaction between non‐thermal plasma and cancerous cells is studied. The short‐lived reactive oxygen and nitrogen species (e.g., hydroxyl radicals, atomic oxygen, nitric oxide) produced by plasma are the main effectors that elicit ICD in melanoma while, surprisingly, persistent species do not. This is demonstrated in vitro using a dielectric barrier discharge plasma system and is validated in a vaccination assay in vivo. Plasma generation of reactive species appears to be dictated by the total energy. Collectively, this work provides fundamental insight into plasma interactions with biological material. Furthermore, it lays the foundation for future development of NTP systems for clinical translation. The addition of plasma systems into the existing arsenal of cancer therapies opens the possibility for new combination strategies for safer and more robust control of cancer.

## 1 Introduction

Recent advances in cancer immunotherapy have led to significant positive impacts on patient survival, especially in patients with cancers previously limited to first‐line treatments.^1^, ^2^ Cancer immunotherapy intends to assist a patient's natural cancer immunity cycle to fight cancer,^3^ and major success has been achieved with checkpoint inhibitors such as anti‐PD‐1/PDL‐1 and anti‐CTLA‐4 therapies.^1^, ^2^ However, the benefits of these treatments have been met with challenges including severe side effects and efficacy in only a subset of patients.^4^, ^5^ Therefore, there is a considerable need to develop new treatment modalities that, in combination with current therapies, may help improve clinical outcomes by supporting different steps of the cancer immunity cycle. The goal of treatments should be to enable an effective, self‐sustaining anticancer response in the patient.

One approach to enhance the initial step of the cycle is to induce immunogenic cell death (ICD) in the tumor. ICD is a form of regulated cell death, characterized by the timely release of “danger signals” known as damage‐associated molecular patterns (DAMPs).^6^, ^7^ Several DAMPs have been linked to ICD (e.g., high‐mobility group box 1 (HMGB1), adenosine triphosphate), the most critical and well studied being surface‐exposed calreticulin (CRT).^8^ CRT on the outer leaflet of the cell functions as an “eat‐me” signal for uptake by dendritic cells.^9^, ^10^, ^11^ ICD inducers have been identified, and novel modalities continue to be explored.^10^, ^11^ One such treatment is plasma generated at room temperature and atmospheric pressure, also known as non‐thermal plasma (NTP).

NTP treatment of mice has been shown to reduce tumor burden and extend survival in different cancer types.^12^, ^13^ The majority of these studies have used NTP for direct tumor cell killing or to induce cell senescence via reactive oxygen and nitrogen species (RONS)‐mediated pathways.^13^, ^14^ Small clinical studies with NTP have only recently commenced for palliative and curative treatment of dermatological diseases, and to date, plasma has been effective with mild to no side effects.^15^, ^16^, ^17^

Beginning in 2015, NTP has been investigated for its potential to induce ICD, and it has been reported to stimulate DAMP emission in multiple cancer cell lines in vitro.^18^, ^19^, ^20^ The first in vivo demonstration of NTP‐induced ICD was performed on Balb/c mice bearing subcutaneous, syngeneic CT26 colorectal tumors.^21^ Tumors treated with a dielectric barrier discharge (DBD) plasma resulted in higher expression of DAMPs (CRT and HMGB1) and increased recruitment of CD11c^+^ and CD45^+^ immune cells into the tumor environment. In combination with a therapeutic vaccine, DBD plasma treatment also enhanced cancer‐specific T‐cell responses.^21^ However, the underlying mechanisms by which NTP elicits ICD are still not fully understood.

When plasma is generated, a complex environment of reactive species, charged particles, neutral molecules, ultraviolet radiation, and electric fields is present and can interact with the biological target. Reactive species produced by the DBD plasma in the presence of oxygen were reported as the major contributors for ICD induction, not the physical components.^20^ However, the exact chemical species that are responsible remain unclear. These include RONS with lifetimes ranging from fractions of a second (e.g., atomic species, radicals) to days and weeks (e.g., hydrogen peroxide, nitrite anion). Knowing which short‐lived and persistent species are required for ICD induction will be critical to the understanding and development of NTP technology for cancer immunotherapy.

To address the above challenges in oncology and the underlying questions in plasma chemistry and biological interactions, we sought to delineate the RONS generated by DBD plasma that are responsible for plasma‐induced ICD. Knowing the species in plasma that are critical to ICD induction will lead to the development of an optimized clinical device. We have chosen to work with melanoma, a deadly disease when it reaches a regional or distant stage with 5‐year survival dropping to 63% and 17%, respectively.^22^ While plasma‐induced ICD has been shown in a colorectal cancer model, establishment of the ICD potential of NTP for melanoma is also crucial, as there are many molecular and physiological differences between cancer types. This disease has also been shown to respond to checkpoint inhibitors in the clinic,^1^, ^2^ making it a relevant model for future translational studies to combine NTP with current immunotherapies to broaden and amplify antitumor immunity.

**Figure 1:**
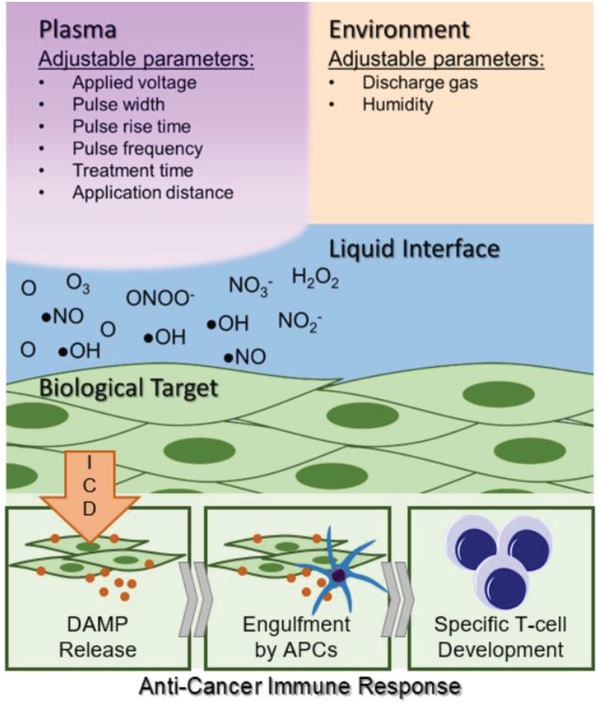
Understanding how non‐thermal plasma interacts with cancerous targets would allow strategic development of a biomedical device for the controlled delivery of RONS to elicit immunogenic cell death. This could assist the initial steps of a patient's cancer immunity cycle and lead to a robust anticancer immune response.

In this study, a thorough examination of the RONS generated by DBD plasma operated at ICD‐inducing regimes was performed. The emission of surface CRT on two melanoma cell lines was used as a surrogate marker of ICD in vitro. Plasma‐generated RONS were measured using electron paramagnetic resonance (EPR) spectroscopy, UV–vis spectrophotometry, and liquid chromatography–mass spectrometry (LC–MS). To further delineate the role of these species on ICD, solutions of RONS were prepared from commercially available sources, used to treat both cell lines, and assessed for their capacity to stimulate CRT. A vaccination assay was performed to validate our findings in vivo. Our results showed that alone, persistent RONS were not sufficient for DBD plasma–induced ICD and highlight the importance of the short‐lived species. Furthermore, we observed that generating the desired RONS with the plasma system is dependent on the plasma treatment energy and not on an individual treatment parameter (e.g., pulse frequency or application time). This study provides crucial information toward our fundamental knowledge of plasma–cell interactions that, through strategic optimization of DBD plasma parameters, would allow us to develop a clinical device for controlled delivery of RONS necessary for ICD (**Figure** **1**).

## 2 Results

### 2.1 DBD Plasma Induces Cell Death and Surface CRT in Melanoma Cells

**Figure 2:**
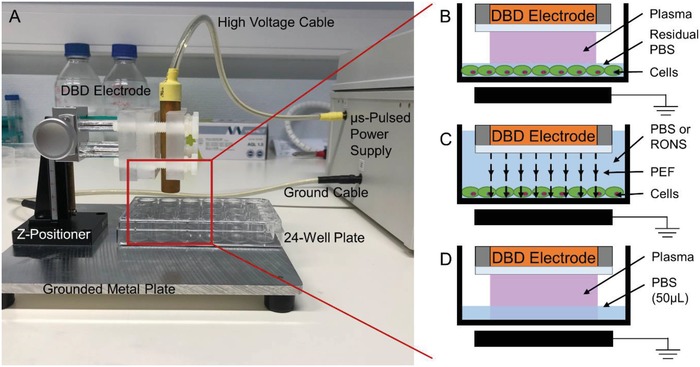
Treatments were performed using a DBD electrode supplied with 17 kV from a microsecond‐pulsed power supply. A) Cells were treated in a 24‐well plate. PBS was used to wash the cells before treatment and was aspirated from the well immediately before exposure to plasma. The following schematics (B–D) are a representation of the zoomed‐in area highlighted in red when the *z*‐positioner was used to fix the DBD electrode 1 mm above the cells for treatment. B) Plasma was generated in this gap directly onto the cells covered by a thin layer of residual PBS (≤2 µL). C) For PEF treatment, the DBD electrode was submerged in liquid, with or without added RONS, positioned 1 mm above the cells, and operated as before. In this case, while the cells still experience microsecond pulses, no plasma was generated due to the high dielectric strength of the liquid. D) For liquid analysis following plasma treatment, the DBD electrode was used to treat 50 µL of PBS, 1 mm above the liquid surface, in 24‐well plates.

To determine whether plasma can induce ICD, we first performed a screening for cell survival and expression of surface CRT in mouse (B16F10) and human (A375) melanoma cell lines. CRT exposure on the cell surface is one of several DAMPs associated with ICD, and has been identified as a critical determinant for anticancer immune responses.^9^ A microsecond‐pulsed DBD plasma treatment system (**Figure** **2**A) was used to generate plasma directly onto the cells after medium was removed and cells were washed with phosphate‐buffered saline (PBS) (Figure 2B). Immediately following DBD plasma treatment, fresh medium was added back into the well.

**Figure 3:**
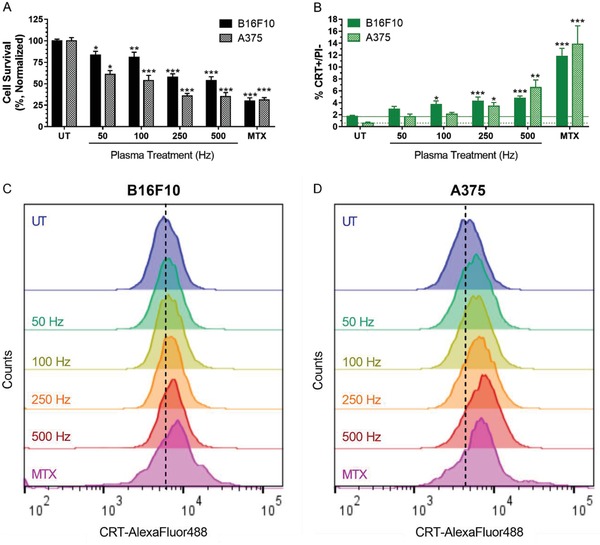
DBD plasma induces cell death and CRT exposure, which increases with higher plasma treatment frequency. MTX (2 µg mL^−1^), a chemotherapeutic ICD inducer, was used as positive control. 24 h after plasma treatment of melanoma cells, cells were collected and analyzed for A) cell survival with a trypan blue exclusion assay and surface‐exposed CRT with immunohistochemistry. B) The percentage of cells that were CRT+ and PI− was quantified with flow cytometry. Histograms of CRT+/PI− cells showed a rightward shift in peak fluorescence. Representative histograms of the C) B16F10 and D) A375 cells are shown here. Data here are represented as mean ± standard error of the mean (SEM) of three to four independent experiments with at least two replicates. Statistical significance of all treatment conditions was compared to untreated. *P < 0.05; **P < 0.01; ***P < 0.001 (generalized linear mixed model).

Cells were treated for 10 s with increasing pulse frequency to deliver higher‐energy treatments. Cells incubated for 24 h with 2 µg mL^−1^ mitoxantrone (MTX), a known chemotherapeutic ICD inducer, were used as a positive control.^9^ Evaluation of cell survival with a trypan blue exclusion assay and an automated cell counter revealed that with increasing plasma pulse frequency, cell survival decreased and plateaus at 250 Hz (**Figure** **3**A). Cells were also double stained with propidium iodide (PI) and a monoclonal CRT antibody. Flow cytometry analysis showed that with higher intensity plasma treatment, the percentage of live CRT‐positive cells (% CRT+/PI−) increased compared to untreated (Figure 3B). Histograms of cells from each treatment group showed a rightward shift in peak CRT fluorescence (Figure 3C,D). The data indicate that there is a general increase in CRT expression, which is not limited to a subpopulation.

It is important to note that surface CRT measured here is only analyzed on PI− cell populations. While dead or membrane‐compromised cells may have higher surface CRT expression after plasma treatment, they also have permeable membranes, resulting in intracellular staining of CRT on the endoplasmic reticulum. Since only surface‐exposed CRT increases immunogenicity and intracellular CRT does not,^23^ it is crucial to delineate them when evaluating ICD in vitro. Therefore, the data presented here act as an indicator of ICD induction, and may be an underestimation of the actual amount of surface CRT on the total cell population. Altogether, our data suggest that plasma is able to elicit cell death and increase immunogenicity of tumor cells in an energy‐dependent manner.

### 2.2 DBD Plasma Generates Short‐Lived and Persistent RONS in PBS

During DBD plasma treatment of cells, PBS was removed from the well and plasma was generated directly onto melanoma cells. However, since the wells were not dried, there remains a residual layer of PBS (Figure 2B), which either interacts with plasma‐generated RONS or creates additional RONS (e.g., via direct electron impact). Due to the close proximity of the liquid to the biological target, RONS generated (including short‐lived species) may influence subsequent biological effect. Therefore, we assessed RONS generated in PBS by DBD plasma at CRT‐emitting parameters. PBS (50 µL) was treated in 24‐well plates (Figure 2D) at the same operating parameters used to treat the melanoma cells. PBS was then immediately collected and analyzed using EPR, LC–MS, or UV–vis spectrophotometry.

#### 2.2.1 Short‐Lived RONS Generated by DBD Plasma (^•^OH, ^•^NO, O/O_3_)

**Figure 4:**
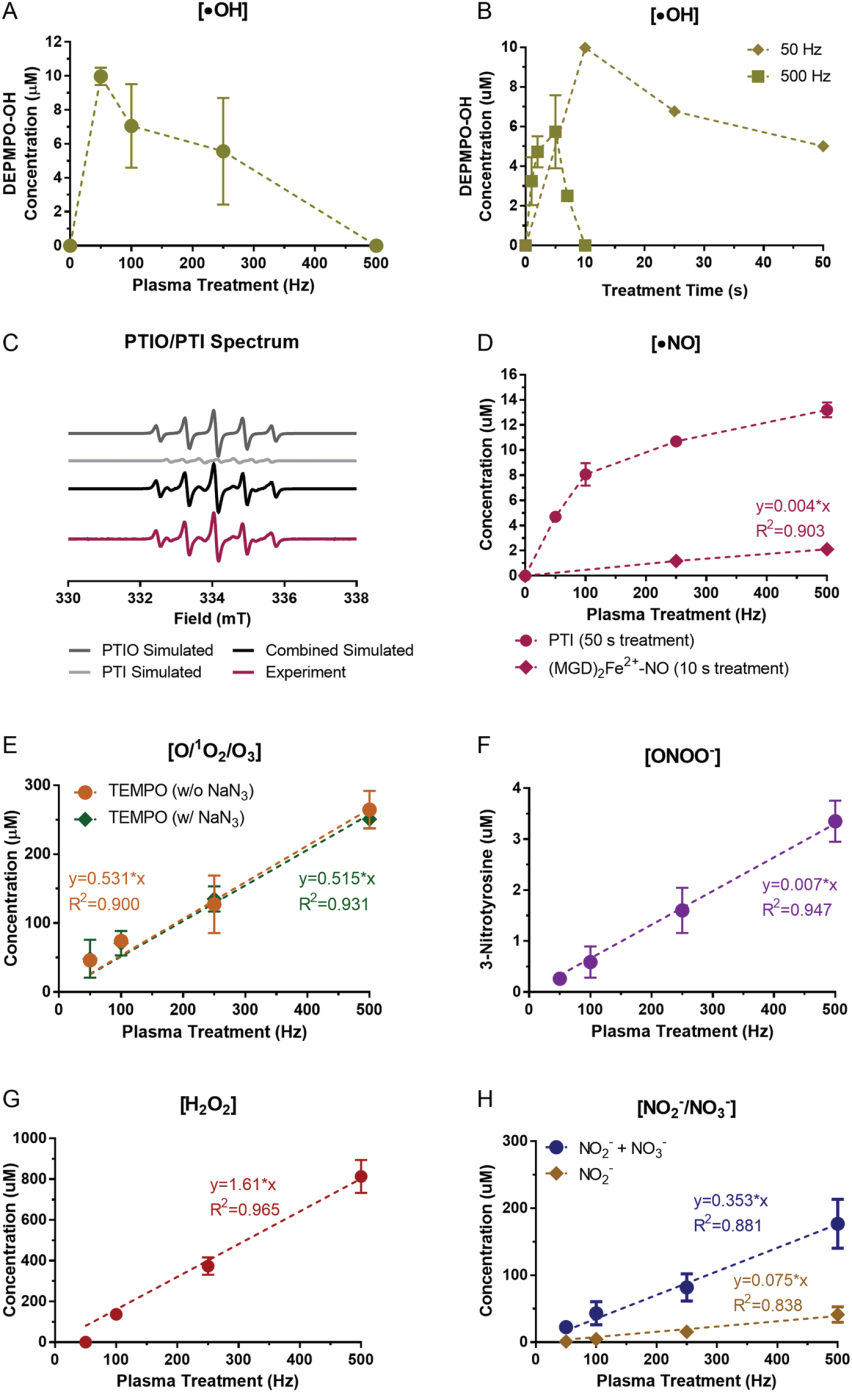
DBD plasma operated at cell treatment parameters generates short‐lived and persistent RONS in liquid. PBS (50 µL) treated by DBD plasma was immediately collected for analysis. Short‐lived species were analyzed with EPR spectroscopy. A) While O_2_^•−^ was not detected with the DEPMPO spin trap, ^•^OH formed the spin adduct DEPMPO–OH that decreased with increasing plasma treatment frequency at fixed treatment time. B) When plasma treatment frequency was fixed and treatment time was changed, DEPMPO–OH initially increased, followed by a decrease, suggesting that DEPMPO–OH is decaying. C) Both the probe (PTIO) and the product (PTI) were monitored simultaneously from the same EPR spectra to measure ^•^NO. The hyperfine values of PTI and PTIO are *a*_N1_ = *a*_N2_ = 0.80 mT and *a*_N1_ = 0.96 mT and *a*_N2_ = 0.44 mT, respectively. D) The formation of (MGD)_2_Fe^2+^–NO from MGD–iron(II) complex was also used to detect ^•^NO. E) O/^1^O_2_/O_3_ was detected using the TEMP spin trip both with and without sodium azide (NaN_3_), an ^1^O_2_ scavenger. Persistent RONS F) ONOO^−^, G) H_2_O_2_, and H) NO_2_^−^ and NO_3_^−^ increased near‐linearly with higher plasma treatment intensity. All experiments testing fixed treatment times were performed with three to five replicates. Data presented here are the mean ± standard deviation; error bars are present but not visible when they are small in relation to the largest graphed mean (<5%).

The concentration of hydroxyl radicals (^•^OH) and superoxide radical anions (O_2_^•−^) in PBS was assessed with the spin trap 5‐diethoxyphosphoryl‐5‐methyl‐1‐pyrroline *N*‐oxide (DEPMPO). A solution of DEPMPO (100 × 10^−3^ m) was prepared in PBS (without calcium, magnesium, or iron) and treated with DBD plasma. ^•^OH reacts with DEPMPO to form the DEPMPO–OH radical adduct, while O_2_^•−^ would form DEPMPO–OOH.^24^ The EPR spectrum following 10 s plasma treatment at all frequencies showed no DEPMPO–OOH radical adduct. However, DEPMPO–OH was detected (Figure S1A, Supporting Information), and decreased with increasing plasma treatment frequency (**Figure** **4**A). When plasma treatment frequency was fixed at 500 Hz and treatment time was changed, DEPMPO–OH increased up to 5 s after treatment, followed by a sharp decrease (Figure 4B). At 50 Hz treatment, DEPMPO–OH decreased with increasing plasma treatment time from 10 to 50 s. Taken together, this strongly suggests that the DEPMPO–OH spin adduct was decaying with increased treatment intensity (higher frequency or longer time). This decay was due to either degradation reactions of the nitroxide group in plasma‐treated aqueous solutions upon which the radical nature is lost^25^ or generation of other RONS (NO*_x_* compounds) that decrease the stability of the adducts.^26^ Therefore, we conclude that while O_2_^•−^ is not produced and/or not delivered to the liquid following DBD plasma treatment, ^•^OH radical is present, but its dependence on pulse frequency and time cannot be determined.

Nitric oxide radical (^•^NO) was monitored in liquid solutions exposed to plasma by two methods. First, we used 200 × 10^−6^ m solution of 2‐phenyl‐4,4,5,5‐tetramethylimidazoline‐1‐oxyl 3‐oxide (PTIO) in PBS. Nitronyl nitroxides such as PTIO react with ^•^NO, forming imino nitroxides, in our case 2‐phenyl‐4,4,5,5‐tetramethylimidazoline 1‐oxyl (PTI).^27^ Both PTIO and PTI were monitored simultaneously, from the same EPR spectra (Figure 4C). The detected concentrationof PTI increased with increasing DBD frequency, similarly to other RONS, though seemingly in nonlinear fashion (Figure 4D). PTI also increased with longer treatment times at 500 Hz until a decrease at 180 s (Figure S2A,B, Supporting Information). This was most likely due to the decay of the nitroxide moiety of PTI and/or reoxidation of PTI back to PTIO at longer exposure times, as we have shown previously.^25^ Thus, these experiments confirmed the presence of ^•^NO in the liquid after DBD plasma treatment, but could only partially be used to assess the degree of frequency/time dependency of ^•^NO production. Subsequently, we used a solution of a *N*‐methyl‐d‐glucamine dithiocarbamate (MGD)–iron(II) complex to detect ^•^NO.^27^ Since the (MGD)_2_Fe^2+^–NO adduct can be formed from interaction of (MGD)_2_Fe^2+^ with NO_2_^−^,^28^, ^29^ we tested its formation by adding NaNO_2_ in concentrations corresponding to that created during plasma treatment. No signal was detected in this case, confirming that all radical adduct formed was due to the reaction with ^•^NO. Large amounts of reducing agent must be added after plasma treatment due to the oxidation of Fe^2+^ to Fe^3+^, resulting in loss of paramagnetic nature of the adduct.^25^ We found that 10 s treatment still allowed detection of the formed radicals under reasonable experimental conditions (volume of added reducing agent, etc.) (Figure S1B, Supporting Information). Although too low to be quantified at 50 and 100 Hz treatments, (MGD)_2_Fe^2+^–NO concentration at 500 Hz was approximately twice that at 250 Hz, suggesting the near‐linear frequency dependency, as was observed with all other RONS (Figure 4D).

The spin trap 2,2,6,6‐tetramethylpiperidine (TEMP) was prepared in PBS at 50 × 10^−3^ m and used to detect atomic oxygen (O), singlet oxygen (^1^O_2_), and ozone (O_3_)^24^, ^29^, ^30^ following DBD plasma treatment. The amine moiety of TEMP was oxidized by these species, leading to the formation of a stable nitroxide 2,2,6,6‐tetramethylpiperidine *N*‐oxyl (TEMPO). The trends of their generation were observed from the intensity of the TEMPO EPR signal (Figure S1C, Supporting Information), though exact concentrations cannot be computed due to the semiquantitative nature of spin trapping.^30^ The relative amount of ^1^O_2_ can be estimated by using a selective scavenger, NaN_3_.^24^, ^29^ At fixed treatment times (10 s), TEMPO concentration increased near‐linearly (*R* ^2^ = 0.900) with plasma treatment frequency (Figure 4E). Generation of the spin adduct also scales near‐linearly with treatment time at fixed frequencies (Figure S3A, Supporting Information). TEMPO was not reduced with the addition of NaN_3_ (Figure 4E), which strongly suggests that ^1^O_2_ is not present in the liquid during treatment. The measured TEMPO is then formed mainly through O and O_3_. Elg et al. have proposed that this reaction in plasma–liquid systems occurs mostly with O, not O_3_, though this would depend on the relative amounts of O and O_3_ produced in each case.^30^

#### 2.2.2 Persistent RONS Generated by DBD Plasma (ONOO^−^, H_2_O_2_, NO_2_^−^, NO_3_^−^)

Peroxynitrite (ONOO^−^) was detected using solutions of l‐tyrosine in PBS as described in past reports: 100 × 10^−6^ m solutions of l‐tyrosine in 2× PBS were treated by DBD plasma, leading to formation of 3‐nitrotyrosine.^31^ The concentrations of 3‐nitrotyrosine were measured using LC–MS with electrospray ionization. We tentatively attribute the formation of 3‐nitrotyrosine to ONOO^−^, though other species that can yield the nitrated product may be present in our system (e.g., ^•^NO_2_ and other nitrogen species). Therefore, the concentrations of ONOO^−^ stated here are possibly an overestimation of the actual amount present. ONOO^−^ increased near‐linearly as well with increasing plasma pulse frequency (*R* ^2^ = 0.947) up to (3.4 ± 0.4) × 10^−6^ m for 500 Hz (Figure 4F).

The generation of hydrogen peroxide (H_2_O_2_) by plasma has been considered a key component of its anticancer properties.^13^, ^32^ Concentration of H_2_O_2_ was measured with UV–vis spectrophotometry (400 nm) from the reaction with potassium titanium(IV) oxalate solution, and the addition of sodium azide (NaN_3_) (details in the Experimental Section).^33^, ^34^ Due to the detection limit of the instrument, the treatment time was increased to 120 s as H_2_O_2_ was undetected at 10 s treatments. H_2_O_2_ concentration increased near‐linearly with plasma treatment frequency at fixed treatment time (*R* ^2^ = 0.965) (Figure 4G). There was no measurable evaporation within the time frames of our treatment conditions. Since H_2_O_2_ generation also scaled near‐linearly with treatment time (Figure S3B, Supporting Information), it confirmed that H_2_O_2_ did not saturate the liquid, and thus the increased exposure time can be used to assess concentrations at various frequencies. H_2_O_2_ concentration after 10 s treatment was extrapolated as 11.4 × 10^−6^, 31.2 × 10^−6^, and 67.8 × 10^−6^ m for 100, 250, and 500 Hz treatments, respectively. At 50 Hz, H_2_O_2_ concentration was still below the detection limit even after 120 s, but is calculated to be 6.7 × 10^−6^ m at 10 s.

The concentration of nitrite (NO_2_^−^) and nitrate (NO_3_^−^) in PBS was determined using the Griess method.^34^ To delineate the amount of NO_2_^−^ from NO_3_^−^, a nitrate reductase enzyme and cofactor were used to reduce NO_3_^−^ to NO_2_^−^ as described in the Experimental Section.^34^ Both species were generated near‐linearly with increasing plasma pulse frequency when treatment time was fixed (Figure 4H), and when pulse frequency was fixed and treatment time was extended (Figure S3C,D, Supporting Information). Several possible reactions could lead to the formation of NO_3_^−^: 1) NO_3_^−^ is formed in the gas and enters the liquid (in ionic or HNO_3_ form), 2) NO_3_^−^ is formed in the liquid from N_2_O_5_ that enters the liquid, and 3) NO_2_^−^ enters the liquid and undergoes oxidation to NO_3_^−^. Therefore, although more NO_3_^−^ was detected in the liquid following direct DBD plasma treatment, it is possible that the initial NO_2_^−^ concentration was higher. This was taken into account in our cellular experiments with RONS solutions, where NO_2_^−^ was used in amounts corresponding to total NO_2_^−^ + NO_3_^−^ concentrations measured here.

#### 2.2.3 Summary of RONS Generated by DBD Plasma in Liquid

In total, ten short‐lived and persistent RONS generated by DBD plasma were analyzed in liquid and their concentrations appear to increase when either plasma pulse frequency or treatment time was fixed and the other was increased (**Table** **1**). This indicates that RONS generation may be most dependent on the total plasma treatment energy and suggests that fine‐tuning the delivered energy can lead to generation of specific RONS for desired biological outcome.

**Table 1:** List of RONS in liquid evaluated after DBD plasma treatment

Plasma treatment [Hz]	Short‐lived RONS (lifetimes <1 s)a)					Persistent RONS (lifetimes ≥1 s)			
	O/O_3_	^1^O_2_	O_2_^•−^	^•^OH	^•^NO	ONOO^−^ [×10^−6^ m]	NO_3_^−^ [×10^−6^ m]	NO_2_^−^ [×10^−6^ m]	H_2_O_2_ [×10^−6^ m]
0	−	−	−	−	−	−	−	−	−
50	+	−	−	*	*	0.3 ± 0.1	21.6 ± 17.2	1.1 ± 0.4	6.7
100	++	−	−	*	*	0.6 ± 0.3	38.6 ± 7.7	4.7 ± 1.9	11.4
250	+++	−	−	*	+	1.6 ± 0.4	66.1 ± 1.7	15.7 ± 1.9	31.2
500	++++	−	−	*	++	3.4 ± 0.4	135.61.8	41.3 ± 11.7	67.8

^a)^ −, Undetectable; +, detectable with quantifiable trends; *, detectable without quantifiable trends.

### 2.3 Persistent RONS Generated by DBD Plasma Alone Do Not Elicit Cell Death without PEF

To test whether persistent RONS generated by DBD plasma (H_2_O_2_, NO_2_^−^, NO_3_^−^, and ONOO^−^) are the main effectors of cell death, exogenous RONS solutions were prepared and added to both melanoma cell lines. Since RONS concentrations reported above were measured in 50 µL of PBS, while PBS is almost entirely removed from the wells prior to direct DBD plasma treatment of cells (Figure 2B), this volume difference must be reconciled. We observed that treatment of cells with 50 µL of PBS in the well diluted the plasma effect, but treatment of cells with 5 µL of PBS remaining did not (Figure S4A, Supporting Information). This is in line with previous studies that reported ≤20 µL of PBS did not dilute the plasma effect on cell viability.^32^ Therefore, we prepared RONS at ten times the concentration and treated the cells with 5 µL for 10 s, as described in the Experimental Section. This most closely replicates the process of direct DBD plasma treatment and is most realistic to the concentration of RONS generated by plasma and experienced by the cells.

**Figure 5:**
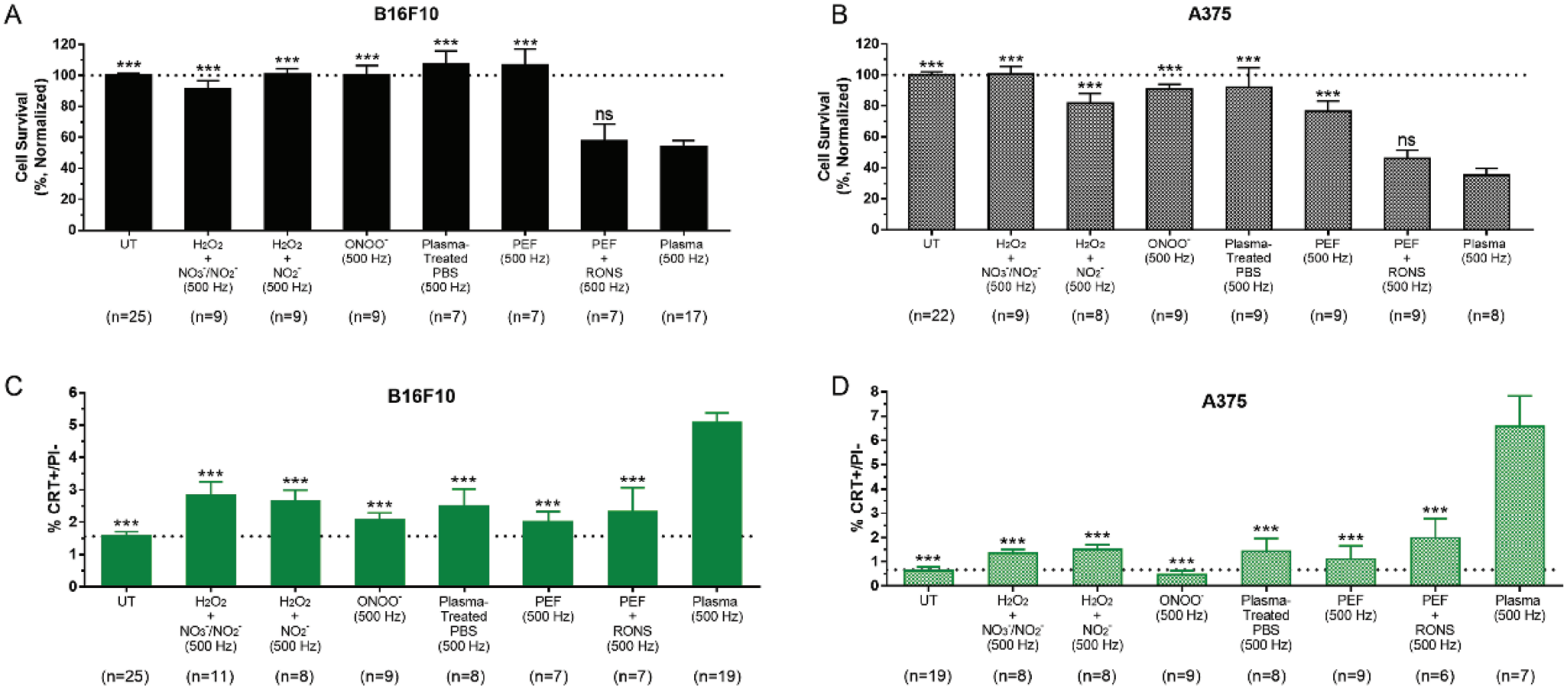
PEF treatment in combination with RONS solution induced cell death but did not elicit surface CRT. Persistent RONS solutions were prepared from commercially available sources at the concentrations determined from 500 Hz DBD plasma treatment. RONS solutions and PEF alone did not elicit cell death in either A) B16F10 or B) A375 melanoma cells similar to that of direct DBD plasma treatment at 500 Hz. PEF treatment in the presence of RONS (PEF + RONS) reduced cell survival but did not increase the DAMP signal associated with ICD, surface CRT, to the same level as DBD plasma treatment at 500 Hz in C) B16F10 or D) A375 melanoma cells. In all graphs, a dotted line is placed at the mean value of the untreated control. Data represented here are mean ± SEM of three to four independent experiments. The total number of observations for each group is shown at the bottom of each column. Statistical significance of all treatment conditions was compared to plasma (500 Hz). ns, *P* > 0.05; ***P < 0.001 (generalized linear mixed model).

As reported above, NO_3_^−^ in the liquid could result from secondary reactions of NO_2_^−^, so two RONS solutions were made based on our measurements: 1) H_2_O_2_ + NO_3_^−^/NO_2_^−^, which accounts for the concentration of all three species, and 2) H_2_O + NO_2_^−^, which assumes all NO_3_^−^ originated from NO_2_^−^. Concentrations of RONS in solution can be found in the Experimental Section. We clearly see that both conditions did not significantly affect cell survival for the melanoma cell lines, as compared to direct DBD plasma treatment (**Figure** **5**A,B). A solution of ONOO^−^ was also prepared in the same way. The stability of ONOO^−^ in PBS was reported to be low, leading to rapid degradation.^31^, ^35^ We tested the stability of commercial peroxynitrite in PBS using a redox probe 3,3′,5,5′‐tetramethylbenzidine (Sigma‐Aldrich, ≥98%, T2885), whose oxidized product can be detected with UV–vis spectrophotometry (Figure S5, Supporting Information).^36^ The results showed that ONOO^−^ was stable in PBS within time frames used here (≤15 s). The solution of ONOO^−^ in PBS was prepared right before treatment and added to the cells immediately to prevent decay. Treatment of cells with exogenous ONOO^−^ also did not affect cell survival (Figure 5A,B).

To further validate whether persistent RONS generated by plasma can elicit cell death, PBS was treated with DBD plasma and then transferred onto cells. 50 µL of PBS was treated for 100 s. Immediately after exposure to plasma, the PBS was added to the cells in the same manner as the RONS solutions described above. Cell survival was also not affected with this treatment group (plasma‐treated PBS), which further highlights that persistent RONS generated here by plasma are not the major effectors of cell death (Figure 5A,B).

When plasma is created with the DBD system, the cells will also experience pulsed electric fields (PEFs) from the high‐voltage DBD electrode. Although electric fields associated with DBD plasma alone do not affect cell death (Figure 5A,B), which is consistent with previous reports,^20^ they may have synergistic effects with the RONS produced by plasma. Therefore, we tested the combination of DBD‐produced PEF and exogenously added RONS. The RONS solution (700 × 10^−6^ m of H_2_O_2_, 1770 × 10^−6^ m of NO_2_^−^, and 35 × 10^−6^ m of ONOO^−^) was prepared immediately before treatment and 1 mL was added to the cells. The DBD electrode was then dipped into the solution and operated as before with the same parameters (Figure 2C). Since the dielectric strength of liquid is much higher than the applied voltage from the electrode, cells in this condition are subjected to PEF without the creation of plasma.

To delineate the effect of PEF alone, the DBD electrode was dipped into PBS instead of the RONS solution. PEF treatment alone did not elicit significant cell death, but when combined with exogenous RONS, cell survival was reduced to that of direct DBD plasma treatment in both the B16F10 (58 ± 11% vs 54 ± 4%, *P* > 0.05) and the A375 (46 ± 6% vs 35 ± 5%, *P* > 0.05) melanoma cell lines (Figure 5A,B). We recognize that applying high‐voltage pulses to the DBD electrode while it is submerged in liquid only produces global electric fields associated with plasma and electric fields from plasma streamers and filaments are not taken into account.^37^ However, our data strongly indicate a synergistic effect between pulsed electric field and plasma‐generated RONS on cell death.

### 2.4 PEF and Persistent RONS Do Not Elicit Surface CRT

Since PEF and RONS had synergistic effects on cell death, we tested whether the immunogenicity of the melanoma cells was also increased by measuring CRT. The effect of PBS volume was first tested in the B16F10 cell line to ensure that 5 µL of PBS did not dilute the DBD plasma effect on surface CRT (Figure S4B, Supporting Information). Again, there was no significant difference between the two conditions. The RONS solutions used for treatment were prepared as described above.

Interestingly, while a combination of PEF and RONS treatment reduced cell survival, it did not elicit equivalent levels of surface‐exposed CRT compared to direct DBD plasma treatment for either melanoma cell lines (B16F10: 2.3 ± 0.8% vs 5.1 ± 0.3%, *P* < 0.001; A375: 2.0 ± 0.8% vs 6.4 ± 1.1%, *P* < 0.001) (Figure 5C,D). Unsurprisingly, PEF alone also did not induce significant CRT on the cell surface above the untreated, which is in line with previous reports.^20^

Neither of the RONS solutions used to treat the melanoma cells elicited significant CRT (Figure 5C,D). However, conditions containing equivalent amounts of H_2_O_2_ (H_2_O_2_ + NO_3_^−^/NO_2_^−^, H_2_O_2_ + NO_2_^−^, plasma‐treated PBS, and PEF + RONS) appeared to increase CRT slightly above untreated in both cell lines at nearly the same amount. Although this change was not statistically significant, it suggests that H_2_O_2_ may affect the immunogenicity of cell death, perhaps at higher concentrations. However, we note that it is not the major contributor to ICD in our DBD plasma treatment regime.

Taken together, our data underline the essential role of short‐lived RONS for affecting ICD. While synergistic effects of PEF and RONS reduce cell survival, they do not elicit CRT required for ICD.

### 2.5 Plasma‐Generated Short‐Lived RONS Are the Major Effectors of ICD

Our in vitro results relied on the detection of CRT as a surrogate maker for ICD, which is only one of several DAMP signals, including heat shock protein 70 (HSP70), HSP90, and HMGB1. However, some studies have shown that certain anticancer agents which are not ICD inducers, are also able to elicit DAMP emission.^38^, ^39^ For example, Dudek‐Perić et al. showed that melphalan, a chemotherapeutic drug, could cause increased surface CRT, HSP70, and HSP90 in melanoma cells, but was not strongly ICD inducing when subject to evaluation in mice.^38^ Additionally, it is still unclear how much CRT expression is needed to elicit an anticancer immune response. Therefore, to address the limitations of in vitro ICD assessment, the current “gold standard” method for confirming ICD induction with a particular stimulus is with a prophylactic vaccination assay where development of an effective, specific T‐cell response can be observed.^7^

**Figure 6:**
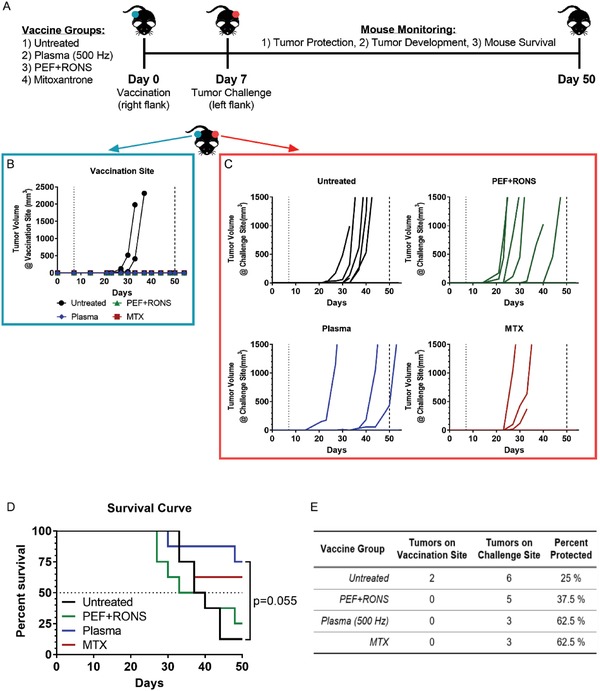
The immunogenic potential of DBD plasma is not due to pulsed electric field and RONS alone, as evaluated using the vaccination assay. A) Syngeneic C57BL/6J mice were vaccinated once and challenged with 10^4^ live B16F10 melanoma cells on the following week (*n* = 8 per group). Tumor volumes were monitored for both B) the vaccination site and C) the challenge site. A dotted line was placed in the graphs on day 7, representing the day of live tumor challenge, while a dashed line was placed on day 50 to represent the predefined end of the study when survival was assessed. All remaining mice were sacrificed together when the final mouse with a tumor reached humane endpoints. D) Mouse survival was plotted for the entire predefined duration of the study (50 d) and the curves were compared with the log‐rank (Mantel–Cox) test. E) Tumor development on both flanks of the mice and percent protection against challenge tumors are listed here.

The vaccine was prepared from B16F10 melanoma cells exposed to DBD plasma (500 Hz), PEF + RONS (RONS: 700 × 10^−6^ m of H_2_O_2_, 1770 × 10^−6^ m of NO_2_^−^, and 35 × 10^−6^ m of ONOO^−^), or MTX (2 µg mL^−1^) in vitro following the same treatment procedures as before. Untreated cells were used as a negative control. After treatment, cells were collected, resuspended in PBS, and incubated for 24 h at 37 °C with 5% CO_2_ to reduce the viability of cells in the suspension (≈12% viable; Figure S6, Supporting Information) and prevent subsequent tumor growth at the vaccination site. This procedure was selected after trying several different methods and optimization processes (Figure S6, Supporting Information). Thirty‐two syngeneic C57BL/6J mice were vaccinated subcutaneously in the right flank (eight mice per group) and challenged with live B16F10 melanoma cells, 7 d later in the contralateral flank (**Figure** **6**A). Tumor development (on both the vaccination and challenge sites), mouse survival, and tumor protection at the end of the study were recorded.

Only 2 of the 32 mice (both from the untreated group) developed tumors at the vaccination site, indicating that the method of preparing the vaccine is relatively safe (Figure 6B). This is important as tumors that develop on the vaccination site contribute to the total tumor volume, thus shortening the duration of the study. Tumors were measured with digital calipers to determine the volume, and mice were sacrificed when tumor burden became too large (total tumor volume >1500 mm^3^) or when tumors began to ulcerate. Of the two mice that developed tumors at the vaccination site, one also developed a tumor at the challenge site, though it was small (Figure S7, Supporting Information).

Tumor volumes at the challenge site were monitored and recorded (Figure 6C). Mouse survival was analyzed at the end of 50 d from the day of vaccination as predefined in our experimental protocol. Only one mouse survived in the untreated group and three mice survived in the PEF + RONS group (Figure 6D). The mice in the plasma group performed much better than those in the untreated control group (*P* = 0.055), and also outperformed the positive control group (MTX) with six surviving mice compared to five, respectively. It is important to note that in the plasma group, there was still one mouse with a tumor that did not reach humane endpoints on day 50. All mice were sacrificed together when the final mouse with a tumor reached humane endpoints, and necropsies were performed to confirm complete tumor protection on the challenge site of the remaining mice (Figure S8, Supporting Information). When mice were vaccinated with untreated cells, only 25% of the population was protected against tumor challenge (Figure 6E). Mice vaccinated with cells treated with PEF + RONS did not improve protection compared to untreated. However, mice inoculated with the plasma‐created vaccine had equivalent protection from tumor challenge to that of our positive control group (MTX) (Figure 6E).

Here, it is important to note that even the untreated control vaccine contained mainly dead cells (≈12% viability). This is an attempt to eliminate tumor growth at the vaccination site, since if the fraction of living cells is too high, neoplastic lesions can develop at the vaccination site.^40^ This would subsequently impact the immunological response at the challenge site and protective immunity cannot be established.^7^ Therefore, although the majority of the cells in the vaccine were killed in all groups (Figure S6, Supporting Information), we have clearly demonstrated that not all forms of cell death resulted in a protective antitumor response (Figure 6C–E).

These data strongly suggest that not only is DBD plasma treatment a *bona fide* ICD inducer for melanoma cells, but the short‐lived RONS are also crucial for stimulating ICD. Here we see that DBD plasma in this treatment regime is more than the sum of the persistent RONS and PEF.

## 3 Discussion

Immunotherapy, whereby the immune system is activated to attack cancer cells, is a major breakthrough in today's cancer treatment. It is generally believed that the cancer immunity cycle can be initiated with cancer cells that die in a way that activates the immune system, called immunogenic cell death.^3^, ^8^ Very recently, NTP has been identified to be an ICD inducer.^21^ However, before this technology can be further developed for clinical use, an in‐depth understanding of how NTP elicits ICD is required.

In this study, we determined the operating parameters of a microsecond‐pulsed DBD plasma to induce ICD in melanoma cells and examined the RONS produced in this regime. This has never been reported before and is an important outcome, with major implications on the plasma treatment modalities that can induce ICD. RONS evaluated here include short‐lived (lifetimes <1 s) and persistent RONS (lifetimes ≥1 s). Solutions composed of H_2_O_2_, NO_2_^−^, NO_3_^−^, and ONOO^−^ at concentrations equivalent to those generated by DBD plasma were prepared and used to treat melanoma cells to further delineate their role. We observed that none of these four species was able to elicit significant cell death or surface‐exposed CRT, a hallmark of ICD (Figure 5). Interestingly, the persistent RONS in combination with pulsed electric fields have synergistic effects on cell death (Figure 5A,B), but this did not elicit CRT expression (Figure 5C,D). It is well known that not all modalities of cell death (e.g., apoptosis, necroptosis, ferroptosis, etc.) are immunogenic^41^; therefore, NTP may be a valuable and unique tool to study fundamental cell death mechanisms.

Determining the importance of short‐lived species for desired biological effect is also critical for the development of clinical NTP devices. In addition to DBDs, another subset of NTP devices for medical applications is atmospheric pressure plasma jets (APPJs). In APPJs, the majority of the plasma is generated remotely, and plasma products are delivered to the biological target via a carrier gas or ionization waves.^42^ Creation of short‐lived RONS with NTP devices can be fine‐tuned by careful manipulation of plasma parameters (e.g., applied voltage, pulse width, pulse rise time, pulse frequency), treatment parameters (e.g., treatment time, application distance), and environmental parameters (e.g., discharge gas, humidity) (Figure 1). In this study, we also investigated the dependence of RONS generation on two parameters in our DBD system. Our data suggest that DBD generation of RONS is not dependent on pulse frequency or treatment time alone. Rather, the RONS are likely generated based on the total number of pulses delivered, which is related to the total delivered plasma energy. Therefore, by controlling the physical properties of plasma generation, we can optimize the cocktail of chemical species required for subsequent biological effects, in this case, immunogenic cancer cell death.

Another modality of NTP use for cancer applications currently being explored is with plasma‐treated liquids (PTLs). Liquid (often buffered saline, cell culture medium, or water) is treated with NTP (typically on the order of minutes) to be enriched with RONS.^43^ The solution is then injected locally in the tumor or perfused through body cavities with tumors. Studies with mouse models have shown that injection of PTL into tumor‐bearing mice delayed tumor growth.^43^ It is important to note that only the persistent species (e.g., H_2_O_2_, NO_2_^−^, NO_3_^−^, potentially ONOO^−^) are present in PTL. Therefore, its anticancer effect may be through a different mechanism than what we observed here. As stated above, we observed that RONS solution treatments containing equivalent amounts of H_2_O_2_ (H_2_O_2_ + NO_3_^−^/NO_2_^−^, H_2_O_2_ + NO_2_^−^, plasma‐treated PBS, and PEF + RONS) increased CRT slightly above untreated, though this change was not statistically significant (Figure 5). Perhaps with longer treatment times (on the order of those used to create PTL), higher CRT expression could be achieved. However, in contrast to direct plasma treatment, short‐lived RONS still do not survive in these solutions, indicating that PTL may be less effective for inducing ICD. While there are still many ongoing studies to work out the fundamental questions and practical issues of using PTL, for now, the use of PTL addresses another important challenge with clinical application of NTP: treatment of nonsuperficial tumors.

Treatment of superficial cancers, such as melanoma studied here, is relatively straightforward, as NTP can be applied directly. However, tumors inside the body may become problematic and limit the use of NTP. One suggested approach is to use NTP in combination with intraoperative procedures. Following surgical tumor excision, NTP could be used to treat the tumor bed or surgical margins to eliminate remaining cancer cells. These studies still need to be performed, and for that, an ergonomic NTP device should be engineered for clinicians. Engineers and physicists are also designing different plasma source geometries for focused and minimally invasive treatment inside the body. This includes an endoscopic device (micrometer diameters) that can propagate plasma up to several meters in length.^44^ With this device, it again becomes important to determine which plasma‐generated RONS will exit the aperture and reach the intended target, as some short‐lived species may be lost along the path of the tube.

Although the vaccination assay has been performed previously in a CT26 colorectal cancer model,^21^ it was met with some limitations. Of the ten mice receiving the plasma‐created vaccine, only three were protected from tumor challenge, while the rest grew tumors, albeit with smaller volumes at the end of the study. In our study, we have spent significant efforts in optimizing our vaccine (Figure S6, Supporting Information). The result is that of the eight mice vaccinated with our NTP‐created vaccine, six mice survived to the end of the study and five were completely tumor free (Figure 6D,E). Furthermore, none of the eight mice developed tumors from the vaccine (Figure 6B). Therefore, not only is this the first report of *bona fide* ICD induction that is relevant for melanoma, a disease with high potential for clinical implementation of NTP therapy, but this is also the first report demonstrating that NTP‐created vaccine can be safely prepared and offers complete protection. This could be further leveraged for plasma‐mediated control of nonsuperficial tumors. We encourage other research groups looking to perform the vaccination assay to follow our protocol, dictated in the Experimental Section and Supporting Information (Figure S6, Supporting Information), for faster optimization, reduction of model animals used, and more comparative results.

It is clear that several hurdles must be addressed before NTP becomes mature for clinical use, and understanding how NTP interacts with cancer cells will provide valuable insight into potential solutions. Ultimately, it is unlikely that NTP alone will be the solution to cancer therapy, but a combination of different therapies may be required. In the past, NTP has been combined with chemotherapy and even a therapeutic cancer vaccine.^12^, ^21^ We also suggest combining DBD plasma with other immunotherapeutic agents, particularly checkpoint inhibitors like PD‐1/PDL‐1 inhibitors, as ICD induction with DBD plasma may have a niche to further improve specific anticancer immune responses. Combination therapies with radiation, chemotherapeutics, and checkpoint inhibitors are ongoing. The success of treatment is partially dependent on finding the proper treatment fractionation scheme for specific cancer types.^45^ Using existing literature and experience with chemoradiotherapy as a basis, effective treatment schedules and combination orders with NTP should be developed and evaluated. However, fundamental differences between radiation therapy and NTP should also be taken into account. For example, while radiation beams can be deeply penetrating, NTP‐generated RONS remain relatively superficial^46^, ^47^ this could limit off‐target effects, but require higher intensity and more repeat treatments. Strategies should also be based on assisting different steps of the cancer immunity cycle or attacking multiple hallmarks of tumor immune evasion.^3^, ^48^

## 4 Conclusion

In summary, we demonstrate that DBD plasma is able to induce *bona fide* immunogenic cell death of melanoma cells and the observed effect is not solely due to the persistent RONS generated (H_2_O_2_, NO_2_^−^, NO_3_^−^, and ONOO^−^). The short‐lived RONS produced by DBD plasma are required. As DBD plasma is highly tunable and treatment is localized, it may have unique advantages over current ICD inducers (e.g., chemotherapeutics, radiation, high hydrostatic pressure, etc.), and should be investigated further with other cancer types.

## 5 Experimental Section

*Experimental Design*: The overall objective of the study was to uncover the mechanism by which non‐thermal plasma elicits immunogenic cancer cell death by evaluating the role of RONS generated. This was accomplished by 1) determining an ICD‐inducing regime of plasma, 2) identifying the RONS generated in that regime, and 3) delineating their effect by comparing direct DBD plasma treatment and treatment with exogenously prepared RONS solutions. Our initial screenings of RONS effect on ICD were performed in vitro on two melanoma cell lines and validated in an in vivo vaccination assay using syngeneic mice.

*Cell Lines*: The B16F10 murine melanoma cell line and the A375 human melanoma cell line were purchased from the American Type Culture Collection. Both cell lines were cultured in complete Dulbecco's modified Eagle medium containing 10% fetal bovine serum, 100 U mL^−1^ penicillin, 100 µL streptomycin, and 4 × 10^−3^ m l‐glutamine. Cells were cultured in a humidified environment at 37 °C with 5% CO_2_.

*Microsecond‐Pulsed DBD Plasma Parameters for Direct Treatment*: A microsecond‐pulsed power supply was purchased from Advanced Plasma Solutions, and a 1.25 cm diameter copper DBD electrode was used for treatment in 24‐well plates. The copper electrode was covered with a 0.5 mm fused‐silica dielectric (Technical Glass) to prevent current arching. The microsecond‐pulsed power supply generated 17 kV pulses with ≈5 µs rise times and ≈1.5 µs pulse widths. The duty cycle was fixed at 100%.

Both cell lines were seeded into 24‐well plates at 3 × 10^5^ cells mL^−1^ (0.5 mL per well) 1 d prior to plasma treatment. On the day of plasma treatment, medium was removed and cells were washed twice with PBS to remove serum and other organics from cell culture medium. PBS from the second wash was left in the well until right before plasma treatment. For DBD plasma treatment, PBS was removed and the DBD electrode was lowered into the well and positioned 1 mm above the cells with a *z*‐positioner (Figure 2A,B). Plasma was then discharged directly on the cells for 10 s at various pulse frequencies (50, 100, 250, and 500 Hz). Following plasma treatment, 500 µL of fresh cell culture medium was immediately added back into the well. Cells were incubated at 37 °C with 5% CO_2_ for 24 h until further analysis. Mitoxantrone dihydrochloride (Sigma‐Aldrich, ≥97%, M6545), a chemotherapeutic used as a positive control, was diluted to a 5 mg mL^−1^ stock solution and prepared into a working solution of 2 µg mL^−1^ in complete medium. Cells were incubated with mitoxantrone for 24 h before collection and analysis.

*Treatment with Pulsed Electric Fields*: On the day of treatment, cells were washed with PBS and 1 mL of PBS or RONS solution (700 × 10^−6^ m of H_2_O_2_, 1770 × 10^−6^ m of NO_2_^−^, and 35 × 10^−6^ m of ONOO^−^) was added to each well immediately before treatment. The DBD electrode was then submerged into the liquid and operated at 17 kV and 500 Hz for 10 s, 1 mm above the cells. This method was to subject the cells to PEF generated from the microsecond‐pulsed power supply and DBD electrode without the production of plasma (Figure 2C). Following 10 s treatment, all the liquid was removed from the well and fresh cell culture medium was added. Cells were then incubated at 37 °C with 5% CO_2_ for 24 h until further analysis.

*DBD Plasma Treatment of Liquid*: For liquid analysis, 50 µL of liquid (PBS or deionized water) was added into a 24‐well plate and distributed evenly across the bottom. The DBD electrode was positioned 1 mm above the surface of the liquid using the *z*‐positioner (Figure 2D). DBD plasma was generated at fixed voltage (17 kV), while the pulse frequency and treatment time were varied.

For preparation of plasma‐treated PBS for treatment of cells, PBS was treated for 100 s at 500 Hz. Prior to finishing the 100 s treatment, PBS was removed from the wells containing cells. All 50 µL of plasma‐treated PBS was then collected and added onto cells and 45 µL was removed. After 10 s treatment with 5 µL of remaining plasma‐treated PBS, 500 µL of complete medium was added back into the well and the cells were incubated for 24 h until further analysis.

*Preparation of RONS Solutions and Treatment*: The solutions of H_2_O_2_, NO_2_^−^, and NO_3_^−^ were prepared from commercially available H_2_O_2_ (Sigma‐Aldrich, ≥30%, 95321), sodium nitrite (NaNO_2_) (Sigma‐Aldrich, ≥97%, 237213), and potassium nitrate (KNO_3_) (Sigma‐Aldrich, ≥99%, P8394) dissolved in PBS (without iron, calcium, and magnesium). ONOO^−^ was prepared from NaOONO (Cayman Chemicals, ≥90% solution in 0.3 m sodium hydroxide, 14042‐01‐4) dissolved in PBS. Four different RONS solutions were prepared

1) H_2_O_2_/NO_2_^−^/NO_3_^−^—H_2_O_2_: 700 × 10^−6^ m; NO_3_^−^: 410 × 10^−6^ m; NO_2_^−^: 1360 × 10^−6^ m
2) H_2_O_2_/NO_2_^−^—H_2_O_2_: 700 × 10^−6^ m; NO_2_^−^: 1770 × 10^−6^ m
3) ONOO^−^—35 × 10^−6^ m
4) H_2_O_2_/NO_2_^−^/ONOO^−^—H_2_O_2_: 700 × 10^−6^ m; NO_2_^−^: 1770 × 10^−6^ m; ONOO^−^: 35 × 10^−6^ m

On the day of treatment, the cells were washed twice with PBS, to follow identical handling procedures with plasma treatment. PBS from the second wash was removed immediately before treatment and 50 µL of RONS solution was added into the well and rocked to ensure even distribution on cells. 45 µL was removed and the remaining 5 µL was left on the cells for 10 s. Following treatment, 500 µL of fresh, complete medium was added to the well and cells were incubated at 37 °C with 5% CO_2_ for 24 h until further analysis. This procedure most closely mimics the process of direct DBD plasma treatment and is most realistic to the concentration of RONS generated by plasma and experienced by the cells.

*Cell Survival Assay*: Cell survival was quantified with a trypan blue exclusion test. Following 24 h incubation, cell supernatant was collected. Cells were then washed with 0.5 mL of PBS and detached with 200 µL of accutase. PBS from the wash was also collected with the cell supernatant. The cell suspension was collected, pooled with their supernatant and PBS wash, and homogenized by pipetting. A 50 µL sample was acquired and equal parts 0.4% trypan blue (Gibco, 15250‐061) was added to the sample. Cell counts were performed using a TC20 Automated Cell Counter (Bio‐Rad). The live cell concentration was recorded, and data were represented as a normalization to control.

*CRT Expression from Cell Lines*: CRT was measured using dual staining of PI and a monoclonal CRT antibody. Following 24 h after incubation, cells were washed with PBS, detached with 200 µL of accutase, and washed twice with 2 mL of FACS buffer (500 mL sheath fluid (BD Biosciences, 342003) + 2 g bovine serum albumin (Sigma, A9418) + 1 g NaN_3_ (Merck, 1.06688.0100) in 100 mL H_2_O). Each sample was split into two vials and one was stained with monoclonal primary rabbit anti‐CRT antibody (Abcam, ab196158) while the other was stained with rabbit IgG, monoclonal isotype control (Abcam, ab199091) for 40 min at 4 °C. Cells were then washed once with FACS buffer. 0.5 µL of PI was added to each sample immediately before being quantified with a flow cytometer. Fifteen thousand events were collected and only the PI− cells were analyzed for surface CRT expression. Data were analyzed and gated using the FlowJo software (FlowJo LLC, version 10). Data were expressed as percent CRT positive after accounting for nonspecific binding with their corresponding isotype. The gating strategy is described in detail in Figure S9 in the Supporting Information.

*Mice and Antitumor Vaccination Assay*: Thirty‐two 8‐week‐old female C57BL/6J mice were purchased from Charles River and housed in a pathogen‐free room at the Animal Center of the University of Antwerp. The sample size of this study (eight mice per group) was chosen using information in the literature^7^ and running an a priori power analysis using G*Power software (version 3.0.10). Input parameters included effect size (large, 0.8), α error probability (0.05), power (0.8), and number of groups (4). A total sample size of 24 was calculated to give an actual power of 0.859. Eight mice were randomly assigned to one of four groups, and housed during the whole of the experiment in four separate cages. Two mice from each group were housed in each cage. Investigators were not blind to the groups.

The vaccines for this assay were prepared from B16F10 melanoma cells exposed to 1) DBD plasma (500 Hz), 2) PEF + RONS (RONS: 700 × 10^−6^ m of H_2_O_2_, 1770 × 10^−6^ m of NO_2_^−^, and 35 × 10^−6^ m of ONOO^−^), or 3) mitoxantrone (2 µg mL^−1^) in vitro, while untreated cells were used as a negative control. After treatment, cells were collected, washed twice with PBS, and resuspended in PBS at 10^6^ cells mL^−1^. Cell suspension was incubated for 24 h at 37 °C with 5% CO_2_ to reduce the viability of the cells and prevent subsequent tumor growth at the vaccination site.

On the day of vaccination, mice were shaved with electric clippers (to help visualize tumors) and injected with vaccine (10^5^ cells in ≈100 µL) on the right dorsal side. After 7 d, each mouse was injected with 10^4^ live B16F10 cells (in ≈100 µL) on the left dorsal side. Tumor size and growth were followed up to day 50 as defined prior to the start of the experiment. Three orthogonal diameters were measured using a digital caliper, and volumes were calculated using (4/3π)*r*_1_ × *r*_2_ × *r*_3_. The humane study endpoint was set to when the total tumor volume exceeded 1500 mm^3^ or if tumors began to ulcerate. All animal experiments were approved by the University of Antwerp Animal Research Ethical Committee (ECD‐dossier 2017‐53).

*Detection of H_2_O_2_*: The H_2_O_2_ concentration was detected using potassium oxotitanate dehydrate (Alfa Aesar, 89620) solution in H_2_O and H_2_SO_4_ (Sigma‐Aldrich, 95–98%, 258105M). Concentration of H_2_O_2_ in plasma‐treated samples was determined by UV–vis measurements performed on a Genesys 6 (Thermo Fischer) spectrophotometer with quartz cuvettes (10 mm light path, 2 mm internal width). Titanium(IV) reagent (0.1 m Ti, 5 m H_2_SO_4_) was prepared by dissolving 0.354 g of potassium bis(oxalato)oxotitanate(IV) dihydrate in a mixture of 2.72 mL of sulfuric acid and diluted to 10 mL with Milli‐Q water. 50 µL of plasma‐treated sample was added to the cuvette and diluted with 150 µL of PBS. 50 µL of sodium azide (NaN_3_) (Sigma‐Aldrich, ≥99.5%, S2002) was added to the cuvette and thoroughly mixed. Afterward, 50 µL of Ti sulfate solution was added and homogenized. Air bubbles in the cuvette were eliminated with a sonicator (Branson 3200 ultrasonic bath) and water droplets were wiped from the cuvette before reading at 400 nm.

*Detection of NO_2_^−^ and NO_3_^−^*: A nitrate/nitrite colorimetric assay kit (Cayman Chemical, 780001) was used according to the provided protocol. To detect NO_2_^−^ only, 50 µL of Griess reagent 1 (sulfanilamide) was added to each sample in a 96‐well plate, and 50 µL of Griess reagent 2 (*N*‐(1‐naphthyl)ethylenediamine) was immediately added afterward. The absorbance wavelength was read with a microplate reader Infinite 200 Pro (Tecan) at 540 nm. To detect NO_3_^−^ and NO_2_^−^, a nitrate reductase mixture (Cayman Chemical, 780010) and an enzyme cofactor mixture (Cayman Chemical, 780012) were added to each sample prior to the addition of Griess reagents. This allowed for the conversion of nitrate into nitrite. The absorbance was measured in duplicates and the samples were prepared in triplicates. The concentrations were calculated based on the obtained calibration curve.

*EPR Spectroscopy Analysis*: 50 µL capillaries (Ringcaps) were used to collect plasma‐treated samples, and a MiniScope MS200 spectrometer (Magnettech) was used to perform the analysis. After each plasma exposure experiment, the samples were immediately placed into a capillary tube. The overall time between exposure and analysis was 1 min. The general EPR parameters were as follows: frequency 9.4 GHz, power 3.16 mW (31.6 mW in case of (MGD)_2_Fe_2_^+^–NO), modulation frequency 100 kHz, modulation amplitude 0.1 mT, sweep time 30 s, time constant 0.1, and sweep width 15 mT. The simulated spectrum was double integrated to determine the concentrations reported here. Simulations were performed using hyperfine values obtained from the literature in the Spin Trab Database (National Institute of Environmental Health Sciences, 2018). EPR calibration was performed using solutions of 4‐hydroxy‐TEMPO (Sigma‐Aldrich, 97%, 176141) as reported elsewhere.^33^ Specific spin traps and other molecules were used to detect RONS in the liquid (**Table** **2**). All recorded experimental EPR spectra and simulations are shown in Figure S1 in the Supporting Information, along with the corresponding hyperfine values used. All fixed‐pulsed experiments presented in the results were performed with three to five replicates unless otherwise specified.

**Table 2:**
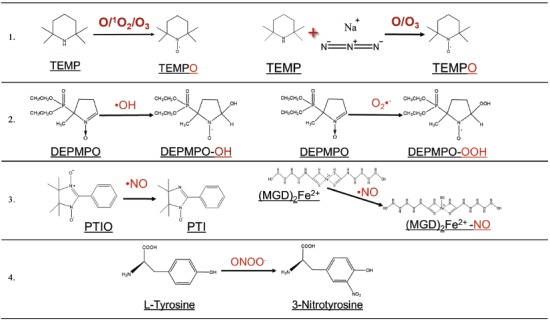
Spin traps and other molecules used in this study to detect RONS in liquid

*Detection of O/^*1*^O_2_/O_3_*: TEMP spin trap was dissolved in PBS (50 × 10^−3^ m) to detect O/^1^O_2_/O_3_ following DBD plasma treatment (Sigma‐Aldrich, ≥99%, 115754). TEMP reacts with these oxygen species to form the spin adduct TEMPO, which can be detected by means of EPR spectrometry. To determine the contribution of O/O_3_, 100 × 10^−3^ m sodium azide (NaN_3_) (Sigma‐Aldrich, ≥99.5%, S2002) was added to the TEMP solution before plasma treatment to quench ^1^O_2_. Therefore, the collected spectrum of TEMPO was a result of the remaining oxygen species.

*Detection of ^•^OH and O_2_^•−^ with EPR Spectroscopy*: DEPMPO spin trap (Enzo Life Sciences, ≥99%, ALX‐430‐093) was dissolved in PBS (100 × 10^−3^ m) to detect ^•^OH and O_2_^•−^. DEPMPO reacts with O_2_^•−^ to produce DEPMPO–OOH while it reacts with ^•^OH to produce DEPMPO–OH. Experiments using 50 Hz pulse frequency were performed once with treatment times of 10 s or more.

*Detection of ^•^NO with EPR Spectroscopy*: The spin probe PTIO (Enzo Life Sciences, ≥98%, ALX‐430‐007) was dissolved in PBS (200 × 10^−6^ m) to detect ^•^NO from DBD plasma treatment. ^•^NO reacts with PTIO to form PTI that can be detected through EPR spectroscopy. When pulse frequency was varied from 50 to 500 Hz, treatment time was fixed at 50 s in order to generate detectable levels of ^•^NO.

The MGD spin trap (Enzo Life Sciences, ≥98%, ALX‐400‐014) was also used to detect ^•^NO. MGD was dissolved in deionized water (20 × 10^−3^ m) and combined with Fe(II)SO_4_·7H_2_O (4 × 10^−3^ m) (Sigma‐Aldrich, ≥99%, 215422). This solution was treated with DBD plasma and Na_2_S_2_O_3_ (100 × 10^−3^ m in deionized water degassed with argon) (Sigma‐Aldrich, ≥98%, 72049) was immediately added to the sample and collected for EPR analysis. When pulse frequency was varied from 50 to 500 Hz, treatment time was fixed at 120 s in order to generate detectable levels of ^•^NO.

*Detection of ONOO* ^−^ *with LC–MS*: Solutions of 100 × 10^−6^ m l‐tyrosine (Sigma‐Aldrich, ≥98%, T‐3754) and 100 × 10^−6^ m diethylenetriaminepentaacetic acid (Sigma‐Aldrich, ≥98%, D1133) in 2 × PBS were exposed to plasma for a given period of time, as described by Wende et al.^31^ The solutions were collected and flash frozen immediately after exposure.

The separation and detection of 3‐nitrotyrosine was done by a Waters ACQUITY ultraperformance liquid chromatograph (UPLC) coupled to a Waters triple quadrupole mass spectrometer (Xevo TQ MS). The used column was a Waters ACQUITY UPLC HSS T3 2.1 mm × 100 mm column (1.8 µm particles), heated to 40 °C. The 9 min gradient was used for separation with A) water containing 0.1% formic acid and B) acetonitrile containing 01% formic acid, at a flow rate of 0.6 mL min^−1^: 0–1.0 min 2% B, 1.0–4.0 min 2% to 18% B, 4.0–5.0 min 18% to 97% B, 5.0–6.0 min 97% B, 6.0–7.0 min 97% to 2% B, and 7.0–9.0 min 2% B. The parameters used in electrospray ionization tandem mass spectrometry in positive mode were as follows: capillary, 0.5 kV; cone, 22 V; extractor, 3 V; source temperature, 150 °C; desolvation temperature, 600 °C; desolvation gas flow, 1000 L h^−1^; cone gas flow, 0 L h^−1^; collision gas flow, 0.15 mL min^−1^; collision energy, 2 V.

A multiple reaction monitoring (MRM) method application was optimized for the detection of tyrosine (transition *m*/*z* 182–136) and 3‐nitrotyrosine (transition *m*/*z* 227–181). For calibration of 3‐nitrotyrosine, eight standard solutions were made ranging from 0 to 10 × 10^−6^ m and analyzed using the MRM method. The samples were diluted to a starting concentration of 10 × 10^−6^ m tyrosine in 95% water and 5% acetonitrile containing 0.1% formic acid.

*Statistical Analysis*: Statistical differences for cell survival and CRT expression were analyzed using the linear mixed model with JMP Pro 13 (SAS software). The fixed effect was the treatment, and the random effects included were the different dates the experiment was performed and the flasks the cells used were split from. The interactions between the treatment and the date as well as interactions between the treatment and the flasks were tested. The random slope model was used when the interactions were significant (*P* < 0.05) and the random intercept model was used in all other cases. The fixed effect tests determine whether there was a significant difference between treatments (*P* < 0.05). When the difference is significant, the Dunnett's test for statistical significance was used to calculate adjusted *P* value compared to the control. A *P* value of <0.05 was considered statistically significant. For all in vitro experiments, treatment conditions were performed in duplicates on the same day and repeated on three separate days as a minimum. The total number of observations for each treatment group is defined in the figure or figure legend. The survival curve of the vaccination study was prepared in Graphpad Prism and compared using the log‐rank (Mantel–Cox) test. A *P* value of <0.05 was considered statistically significant. All figures were prepared in Graphpad Prism (Graphpad Software). For all chemical species, a nonlinear regression was used to determine the best‐fit line and *R* ^2^ value with a *y*‐intercept constraint at zero. Analysis was performed and figures were prepared in Graphpad Prism (Graphpad Software). No data were excluded.

## Conflict of Interest

The authors declare no conflict of interest.

## Supporting information

Supplementary
