# Supplementary material for: Non‐Thermal Plasma as a Unique Delivery System of Short‐Lived Reactive Oxygen and Nitrogen Species for Immunogenic Cell Death in Melanoma Cells
Source: Adv Sci (Weinh). 2019 Jan 28;6(6):1802062. doi: 10.1002/advs.201802062 (PMC6425452; doi:10.1002/advs.201802062)
Supplement: Supplementary file 1 — Supplementary [file ADVS-6-1802062-s001.pdf]

Copyright WILEY-VCH Verlag GmbH & Co. KGaA, 69469 Weinheim, Germany, 2019.

ADVANCED SCIENCE
Open Access

Supporting Information

for *Adv. Sci.,* DOI: 10.1002/advs.201802062

Non-Thermal Plasma as a Unique Delivery System of Short-Lived Reactive Oxygen and Nitrogen Species for Immunogenic Cell Death in Melanoma Cells

Abraham Lin, Yury Gorbanev, JoeyDe Backer, JintheVan Loenhout, WilmaVan Boxem, Filip Lemière, Paul Cos, Sylvia Dewilde, Evelien Smits, and Annemie Bogaerts*

Copyright WILEY-VCH Verlag GmbH & Co. KGaA, 69469 Weinheim, Germany, 2019.

Supporting Information

Title: Non-Thermal Plasma as a Unique Delivery System of Short-Lived Reactive Oxygen and Nitrogen Species for Immunogenic Cell Death in Melanoma Cells

Abraham Lin*, Yury Gorbanev, Joey De Backer, Jinthe Van Loenhout, Wilma Van Boxem, Filip Lemière, Paul Cos, Sylvia Dewilde, Evelien Smits, Annemie Bogaerts

EPR spectra and simulations

The EPR spectra of the spin adducts were collected for the different short-lived RONS and simulated to determine the relative concentration (**Figure S1**).

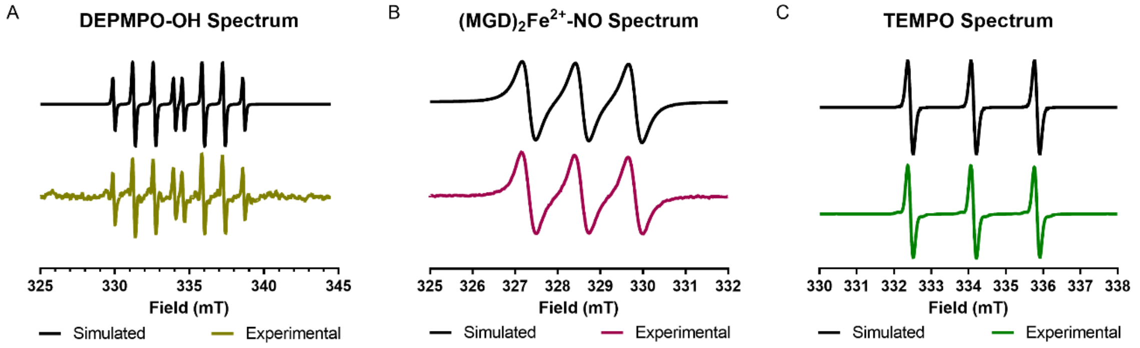

Figure S1. Simulated EPR spectrum of spin adducts were compared to experimental spectrum. (**A**) DEPMPO-OOH was not detected while DEPMPO-OH was. The hyperfine values used were a_N=1.38 mT, a_H=1.30 mT, and a_p=4.65 mT. (**B**) The $(MGD)_2Fe^{2+}$-NO hyperfine value was a_N=1.25 mT. C) TEMPO was simulated with hyperfine values of a_N=1.7 mT.

Decay of PTI and PTIO with increased plasma treatment time

Since PTI concentration did not increase linearly with higher plasma treatment frequency (**Figure 4D** in main text), we investigated whether this was indeed due to a difference in the kinetics of •NO generation by DBD plasma or whether the spin probe and the reactant was decaying. The PTI and PTIO spectrum was monitored simultaneously from the same EPR spectra. When plasma pulse frequency was fixed at 500 Hz and treatment time was increased from 10 to 180 s, we observed an increase up to 120 s, followed by a decrease (**Figure S2**). This is observed in the PTI peaks (marked with asterisks) of the EPR spectra (**Figure S2A**)

and has also been graphically represented by determining concentration from peak intensity (**Figure S2B**). Since we do not see a significant increase at higher treatment times, this suggests that either PTIO decays quicker than PTI is formed, or PTI decays almost at the same rate as it is formed. Altogether, this confirms that the non-linear generation of PTI in PBS from DBD plasma treatment is not due to a difference in kinetics, but due to the degeneration of PTI and PTIO.

A

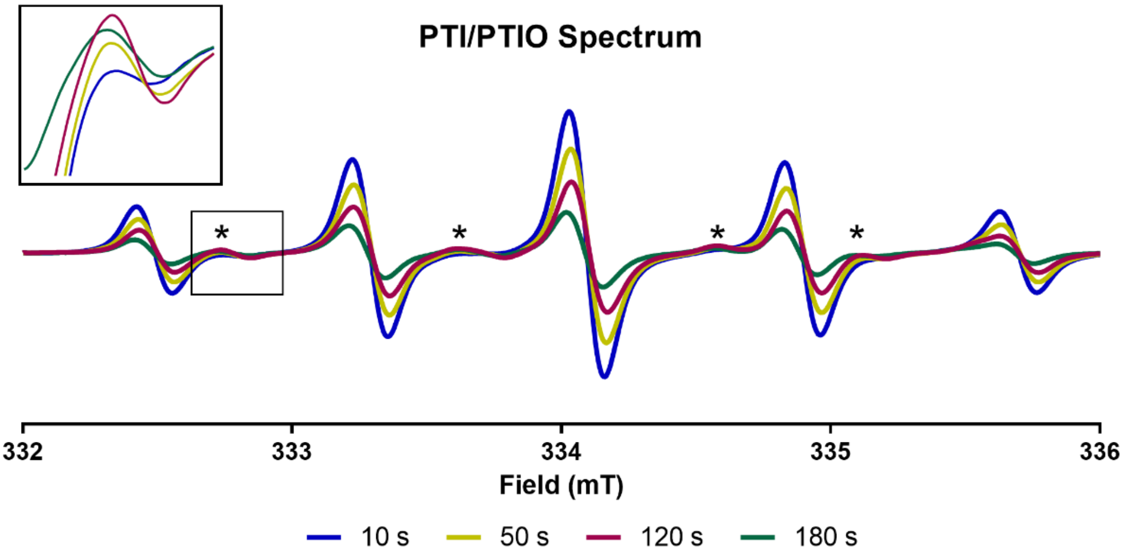

B

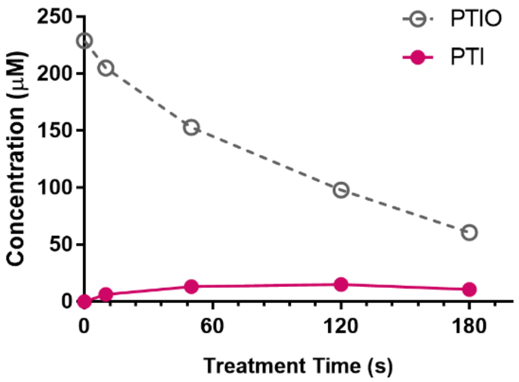

Figure S2. Both PTIO and PTI decay with increased plasma treatment time. (**A**) The asterisks (*) on the spectrum represent the peaks of PTI. The hyperfine values of PTI are $a_{N1}=a_{N2}=0.8$ mT and the hyperfine values of PTIO are $a_{N1}=0.96$ mT and $a_{N2}=0.44$ mT. (**B**) The concentration of both PTIO and PTI decay over time.

Time dependence and pulse dependence of DBD plasma-generated RONS

To determine the dependence of treatment time on DBD plasma-generated RONS in PBS, the plasma pulse was fixed at low and high frequencies, and 50 μL of PBS were treated for a range of times (**Figure S3**). No saturation of any species was observed within the time ranges used and the generation of RONS increased near-linearly with time. Treatment times used here were chosen to deliver the same total number of plasma pulses when frequency was altered (e.g. treatment at 50 Hz for 100 s and treatment at 250 Hz for 20 s both deliver a total of 500 pulses of plasma). Combined with the results when treatment time was fixed at 10 s and pulse frequency was varied between 50 Hz and 500 Hz (**Figure 4** in main text), our data strongly suggest that the generation of RONS in liquid is dependent on the total number of delivered plasma pulses and not on one specific parameter (treatment time or pulse frequency) alone.

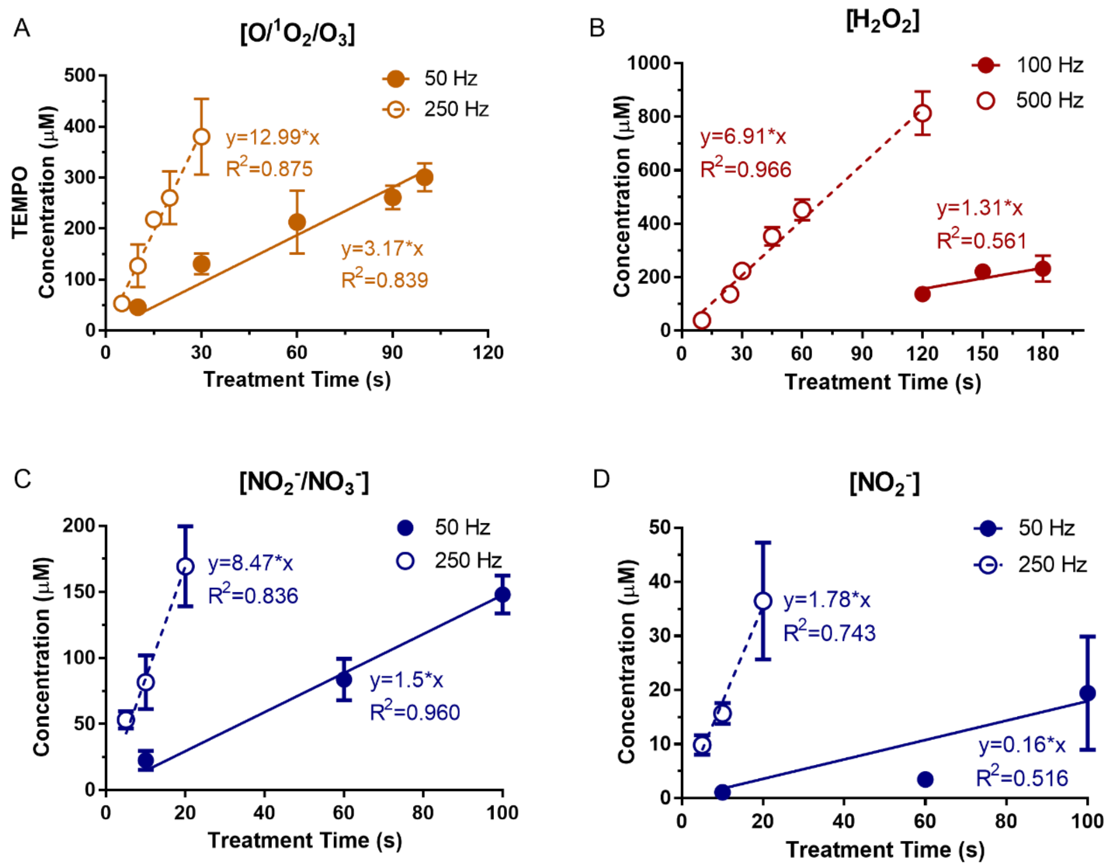

Figure S3. DBD plasma at fixed pulse frequencies generates RONS in PBS near-linearly with treatment time. At both low and high frequency treatments, concentrations of (**A**) H_2O_2, (**B**) TEMPO (indicative of $O/^1O_2/O_3$), (**C**) NO_2^-, and (**D**) both NO_2^- and NO_3^- did not saturate at higher treatment times but increased near-linearly.

Minimum PBS volume present in treatment without diluting DBD plasma effect on cells

All the RONS measurements in liquid in this study were performed on 50 µL of PBS that was treated with DBD plasma in 24-well plates. However, when melanoma cells were treated with DBD plasma at the same treatment parameters, only a residual layer of PBS (~2 µL) is present. Therefore, in order to prepare and treat cells with RONS solutions with concentrations equivalent to that experienced by the cells under plasma treatment, this difference in volume must be reconciled. Both cells lines were treated by DBD plasma at 500 Hz for 10 s with 1) all the PBS removed from the well (only residual remaining, ca. 0 µL), with 2) only 5 µL remaining, and with 3) 50 µL remaining. Cell survival was monitored at 24 h with the trypan blue exclusion assay, as explained in the Methods section. While there is

not a significant difference in cell survival between cells treated with 0 µL or 5 µL of PBS remaining, we observed that when the cells were treated with 50 µL of PBS, cell survival increased by approximately 20% (**Figure S4A**). Therefore, it is clear that treating cells with 50 µL of PBS in the well will dilute the plasma effect, but having 5 µL present will not. We also confirmed that 5 µL also did not affect the plasma effect on CRT emission in the B16F10 cell line (**Figure S4B**). Altogether, this data informed us that the RONS solutions to be used for treatment of cells should be prepared at 10x's the measured concentration in 0.1x's the volume (5 µL) to most accurately reflect DBD plasma treatment and allow for comparison.

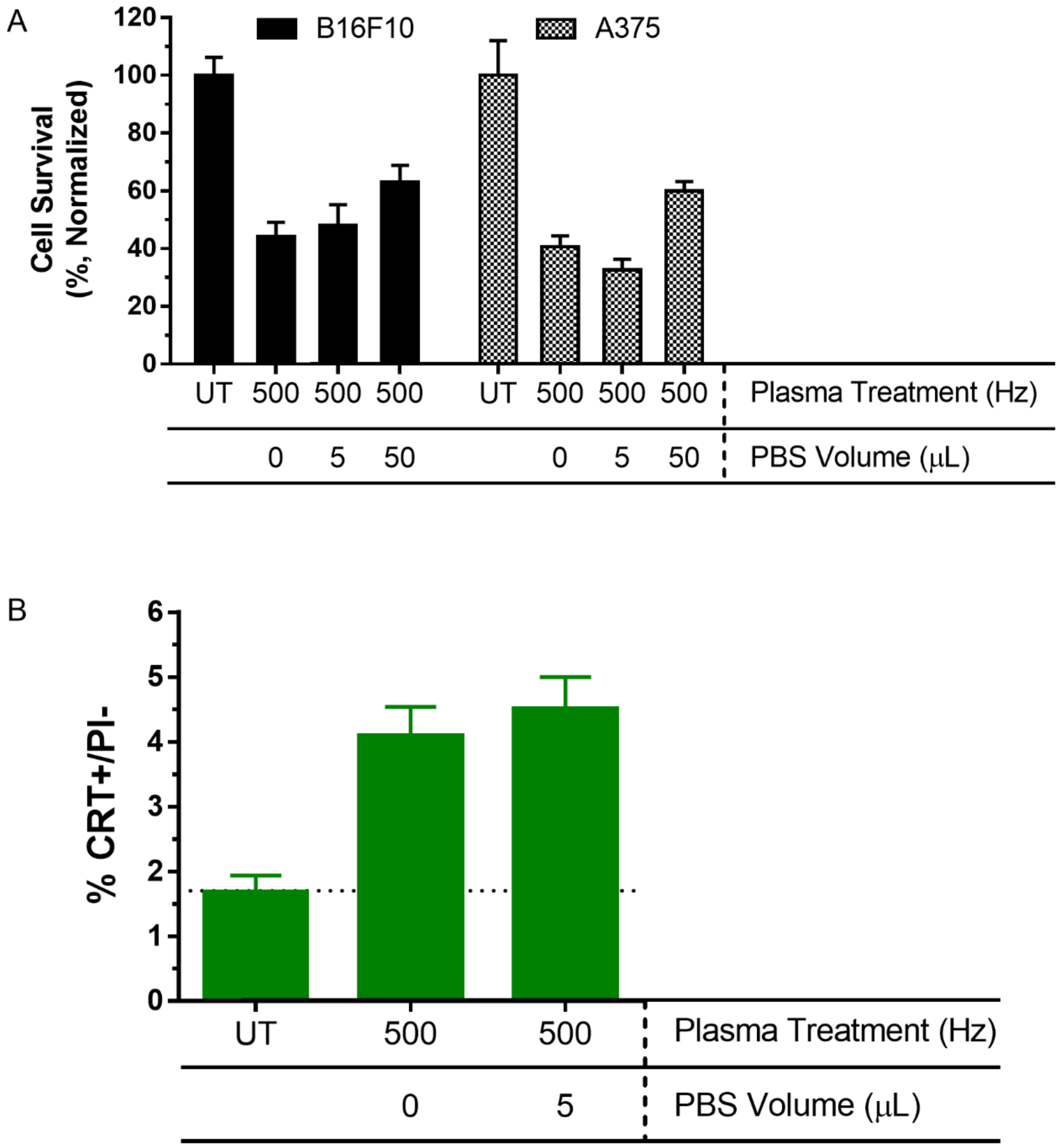

Figure S4. PBS (5 μL) present during DBD plasma treatment of cells did not dilute the effect on cell survival or surface-exposed CRT. Cells were treated with only residual PBS (0 μL), only 5 μL remaining, or with 50 μL in the well and (**A**) cell survival and (**B**) CRT exposure from B16F10 cells was compared to that of the untreated (UT) 24 h later. A horizontal dotted line indicates the mean value (% CRT+/PI–) from untreated cells.

Stability of $ONOO^-$ in PBS at Room Temperature

The stability of $ONOO^-$ under experimental conditions was tested using a colourless redox probe 3,3′,5,5′-tetramethylbenzidine (TMB) (Sigma-Aldrich, ≥98%, T2885), whose oxidized product can be detected with UV-Vis spectrophotometry. $ONOO^-$ solutions were prepared as follows. Commercially available peroxynitrite (Cayman Chemical, ≥90%, 81565) was used to prepare a 40 μM $ONOO^-$ solution in PBS. This solution was incubated at various time

points at room temperature before addition of TMB in an ethanol/water mixture (EtOH:H_2O, 1:1). 1 mL of 2 mM TMB solution was mixed with 1 mL of NaOONO/PBS solution, and the pH was immediately brought to 3.9-4 using 1 M HCl. The solution was allowed to incubate for 30 min in the dark, after which the absorption was measured at 655 nm, corresponding to the oxidation product of TMB. Therefore, the degradation of $ONOO^-$ in PBS could be determined by monitoring the concentration of oxidized TMB after the two solutions were mixed.

The concentration of $ONOO^-$ as a function of its residence time in PBS prior to the addition to TMB shows that while there was no significant decay of $ONOO^-$ within 10 s, this species completely degrades within 10 min (**Figure S5A**). The calibration of this method was performed by mixing 2 mL of 1 mM TMB in EtOH:H_2O (1:1) at pH 4 with various amounts of commercially available $ONOO^-$ (**Figure S5B**). The oxidised product of TMB was stable in the analysed solutions at room temperature for at least 2 hours (**Figure S5C**). No oxidation of TMB was observed with the degradation products of peroxynitrite (e.g. H_2O_2, HNO_2, HNO_3) or NaOH (present in the commercial NaOONO) used in concentrations up to 100 uM (data not shown).

The results show that within timeframes used in our experiments where cells were treated with RONS solution containing $ONOO^-$ ($\leq$ 15s, shown as a vertical dotted line in **Figure S5A**), only approximately 10% of $OONO^-$ decayed. This validates our method of comparing RONS treatment with DBD plasma treatment and suggests that our biological data is a reliable reflection of the $ONOO^-$ effect.

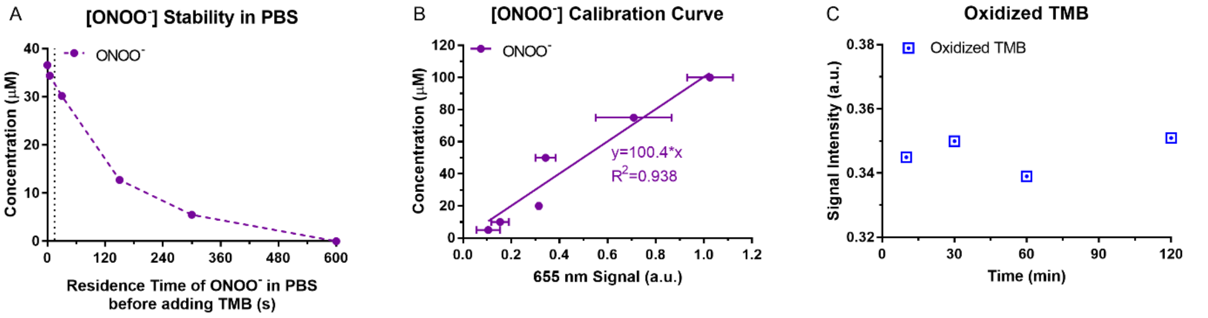

Figure S5. $ONOO^-$ was stable in PBS within time frames used in cell treatment studies. (**A**) Solutions of $ONOO^-$ were prepared in PBS and incubated at room temperature before addition of TMB. Oxidized TMB was then measured with UV-Vis spectrophotometry to determine the amount of $ONOO^-$ degradation. Within 15 s (represented as a vertical dotted line), the $ONOO^-$ decay was approximately 10%. (**B**) The concentration of $ONOO^-$ was determined from a calibration curve where oxidized TMB was measured after addition of various amounts of commercially available $ONOO^-$. (**C**) The product of $ONOO^-$ and TMB, oxidized TMB, was stable in PBS at room temperature for up to 2 h.

Vaccine preparation and delivery optimization

Before attempting a full vaccination assay, we first ran a small pilot study to assess the safety and efficacy of the B16F10 tumor cell vaccine. In several studies in literature, tumors grew at the vaccination site. This would contribute to the overall tumor burden which would then require the mouse to be sacrificed before the vaccination affect could be observed. Therefore, this study would help determine a safe way to prepare the vaccine to extend the mouse survival time to see an immunological effect (~50 days). 12 mice were used in this study and divided into four groups (**Table S1**). The mice received B16F10 cells that were either exposed to 500 Hz plasma or not (untreated control). Both groups were then split further into two groups: with or without one freeze and thaw cycle in liquid nitrogen. The freeze-thaw cycle was performed immediately after cell collection and resuspension into injection volume in 1.5 mL Eppendorf tubes to reduce the viability of injected cells. This was intended to eliminate or delay background tumors at the vaccination site. 100 μL of the vaccine was injected into the right flank of each mouse (10^5 cells/mouse). 7 days later, the mice were challenged with live 10^5 live B16F10 cells on the contralateral flank and the tumor volume was monitored as described in the methods. In both control groups (with and without freeze-thaw cycle), two mice reached humane endpoints and had to be sacrificed. One mouse in the

plasma-treated group without freeze-thaw was also sacrificed. All remaining mice in every group were sacrificed on day 21, 30 days before our intended study endpoint (**Figure S6A**). It was clear that 1 freeze-thaw cycle was not enough to reduce the viability of the whole-cell vaccine for the experiment to reach the desired endpoint (50 days). Therefore, we aimed to further optimize our vaccine preparation and delivery method.

Table S1. Four groups were used for the pilot vaccination study

Groups	Plasma Treatment	Freeze/Thaw Cycle
1. Untreated Control	No	No
2. Plasma Only	Yes	No
3. Freeze-Thaw Control	No	Yes
4. Plasma+Freeze-Thaw	Yes	Yes

We tried several different methods to reduce the viability of cells in our whole-cell vaccine, including: 1) sonication, 2) addition of lysis buffer with protease inhibitor, 3) increased freeze-thaw cycles, and 4) incubation in PBS. Sonication in the (Branson 3200 ultrasonic bath) for 3 to 5 min did not reduce cell viability. The addition of lysis buffer and protease proved to be impractical and also introduced additional chemicals into our vaccine and therefore was not used further. To determine the number of freeze-thaw cycles required to reduce cell viability, B16F10 cells were collected and resuspended in PBS (10^6 cells/mL) and aliquoted into cyrotubes (1 mL/tube). The tubes were then submerged into liquid nitrogen for 10 seconds and thawed in the warm water bath (37°C) to complete 1 freeze-thaw cycle. Cells from the tube were then seeded into 24-well plates with 500 μL of complete media and incubated at 37°C with 5% CO_2. Images were taken on day 3 at 20x with the EVOs bright-field microscope. We observed that cells continued to grow after 1 freeze-thaw cycle, but did

not after 3 and 5 cycles (**Figure S6B**). However, there appears to be a lot of cellular debris following 3 and 5 freeze-thaw cycles.

Another method we tested was to incubate cells in PBS for 24 h in the injection volume (**Figure S6C**). The cells were collected, washed twice with 1 mL of PBS, and aliquoted into Eppendorf tubes at 10^6 cells/mL (1 mL/tube). The tubes were then incubated at 4°C or 37°C. 24 h after incubation, 10 μL were collected and analysed with a trypan blue exclusion assay as described in the methods. The rest of the cell suspension was added into 6 well-plates and incubated overnight at 37°C with 5% CO_2. Bright-field images (10x) were taken the next day to assess cell viability. 24 h after incubation in PBS, cells at 37°C had a reduced viability compared to those at 4°C (12% vs 95%, respectively) (**Figure S6D**). Counting slides of cell suspension stained with trypan blue was also visualized using the EVOs bright-field microscope (20x). Furthermore, cells incubated at 4°C continued to grow, 48 h later, while the majority of those incubated at 37°C did not (**Figure S6D**).

Since incubation in PBS significantly reduced cell viability and from the bright-field images, cells appear to retain their structure more compared to freeze-thaw cycles, we proceeded to the full animal study using this method to prepare the vaccine. Moreover, we incubated the vaccine in PBS in 24-well plates instead of Eppendorf tubes so that surviving cells would attach to the bottom of the plate. Collection of the supernatant would then further exclude the live population.

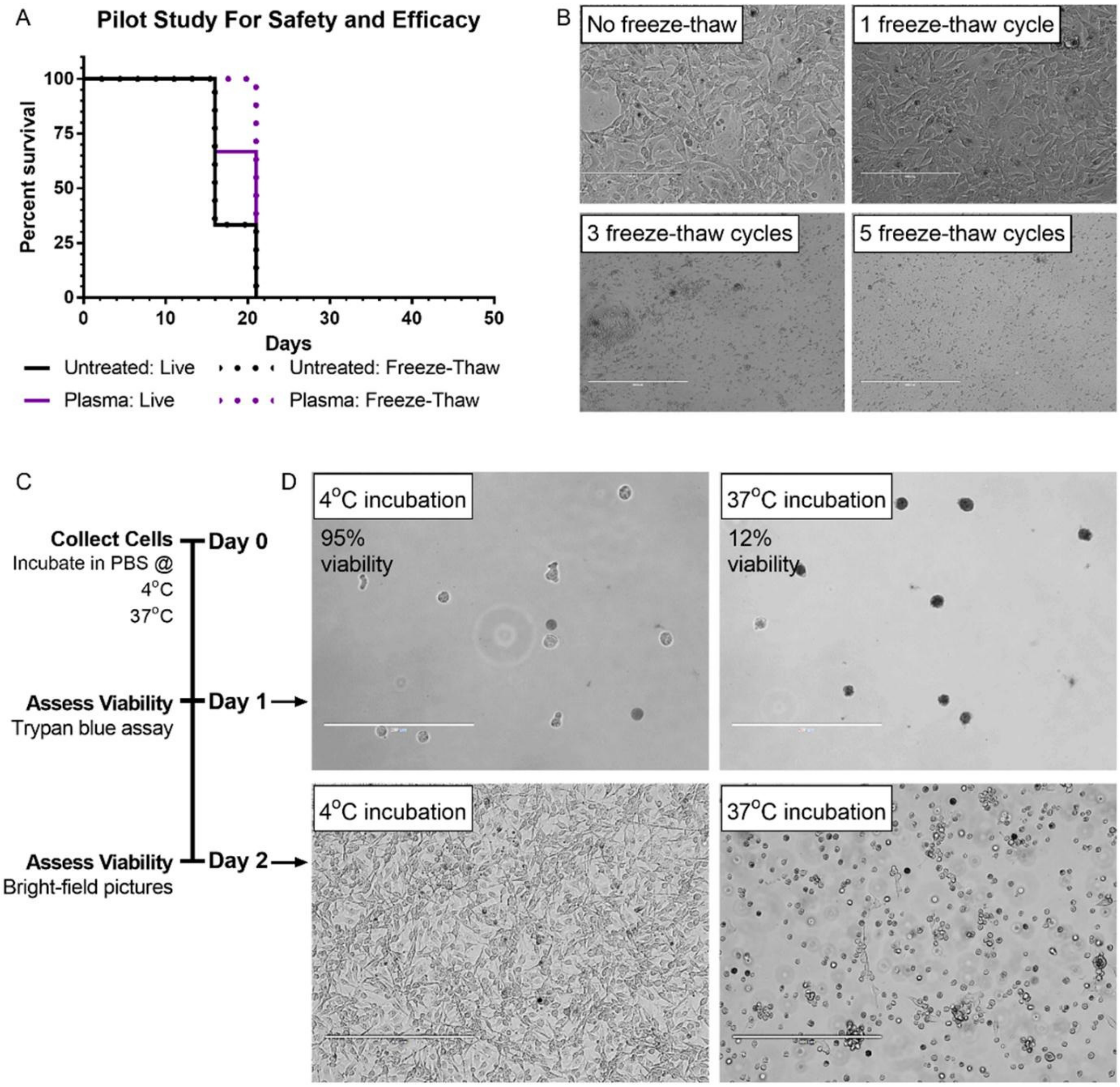

Figure S6. The method of preparing the vaccine was optimized. (**A**) A small pilot study (n=12) was performed to determine whether the experiment would reach the desired endpoint (50 days). Mice were vaccinated in the right flank with B16F10 cells (10^5 cells/mouse) either treated or untreated with DBD plasma (500 Hz). In one of the untreated groups and in one of the plasma groups, the vaccine underwent 1 freeze-thaw cycle in liquid nitrogen before injection. This was intended to eliminate or delay tumor growth at the vaccination site. The survival curve shows that no animal survived past 21 days. (**B**) Images taken on day 3 with the EVOS bright-field microscope (20x) showed that cells that underwent 1 freeze-thaw cycle (top right) continued to grow similar to that which did not (top left). Cells that underwent 3 (bottom left) or 5 (bottom right) freeze-thaw cycles, however, had significant reduction in cell viability and increase in cell debris (scale bar=200 µm). (**C**) We also attempted to reduce viability of cells in the whole-cell vaccine by incubating cells in PBS 24 h before injection. Cells were collected, washed twice with PBS, and resuspended at 10^6 cells/mL. Cells were aliquoted into Eppendorf tubes and incubated for 24 h in either 4°C or 37°C. On the next day (day 1), 10 µL of cell suspension was collected and the viability was analysed using the trypan blue exclusion assay. The remaining cell suspension was seeded into 6-well plates and 500 µL of complete cell media was added. The cells were incubated at 37°C with 5% CO_2 overnight and imaged with the EVOS on day 2 to further assess viability. (**D**) Viability remained high in cells incubated at 4°C (top left) compared to that of cells incubated for 24 h at 37°C (top right). The dark colored cells in the images represent trypan blue positive cells, which were quantified with an automated cell counter, described in the methods. Images of the slides are taken with the EVOs at 20x (scale bar=200 µm). Incubation of the remaining cell suspension showed that cells first incubated at 4°C continued to grow and proliferate (bottom left), while the majority of those initially incubated at 37°C did not (bottom right). Images are taken with the EVOs at 10x (scale bar=400 µm).

Images of mice with tumors on the vaccination site

Two mice developed tumors at the vaccination site and had to be sacrificed before any tumors were measured on the challenge site. Necropsy was performed post-mortem to determine whether tumors had formed on the challenge site. One mouse had developed a small tumor on the challenge site while the other did not (**Figure S7**).

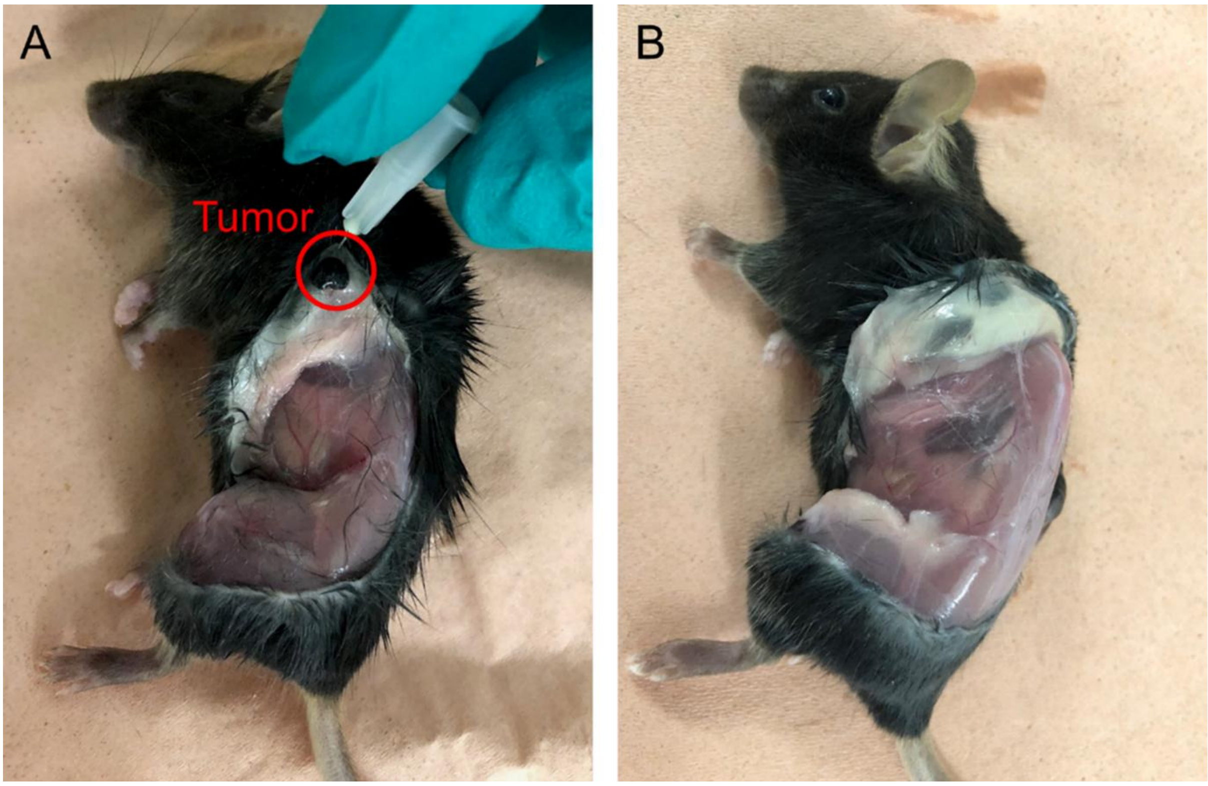

Figure S7. Necropsy of the mice that developed tumors at the vaccination site was performed when tumor burden exceeded the defined humane endpoints. (**A**) One mouse developed a tumor (circled in red) at the challenge site while (**B**) the other did not (dark areas on the mouse were checked and were a result of darker skin pigmentation).

Images of the tumor challenge site on mice following necropsy at the end of the study

All mice were sacrificed together on the day when the final mouse with a tumor reached humane endpoints. Necropsies were performed to confirm that remaining mice were indeed tumor-free after live cancer cell challenge (**Figure S8**). Apart from the mouse that required euthanization (**Figure S8C**, tumor circled in red) all other mice did not have tumors at the challenge site.

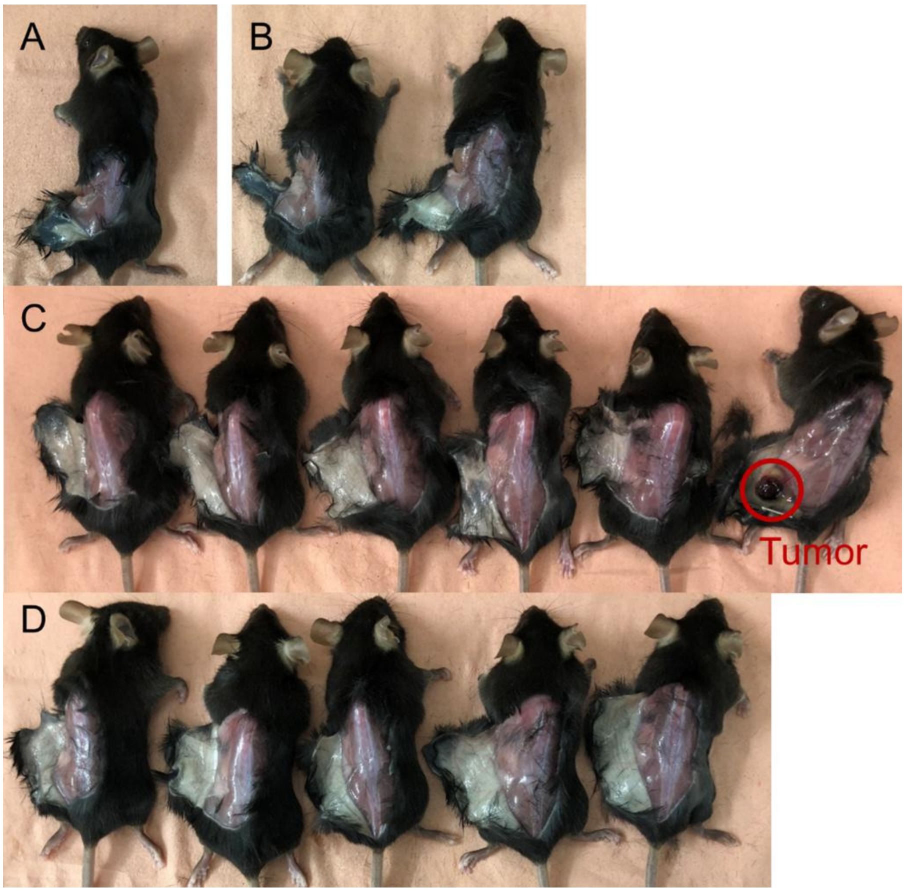

Figure S8. All remaining mice were sacrificed and necropsied together when the final mouse with a tumor reached humane endpoints. The (**A**) Untreated group, (**B**) the PEF+RONS group, (**C**) the Plasma (500 Hz) group, and (**D**) the Mitoxantrone group all showed no tumor development at the live cancer challenge site apart from the (**C**) final mouse with a tumor which is circled in red.

Gating strategy for surface CRT identification

Following CRT and PI dual staining, flow cytometry was performed on cell suspensions. A total of 15,000 events were collected for each sample and data were exported. All gating and analysis was performed in the FlowJo software. First, the cell population was identified and gated out from the debris based on the forward scatter (FSC-A) and side scatter (SSC-A) plots. Within this population, the PI negative (PI–) population was gated to exclude all dead and permeable cells from the population. Finally, within the PI– population, the CRT positive (CRT+) gate was made. This was based on the unstained and untreated isotype-stained population. Once the gates were fixed, they were applied to all treatment groups within the experiment before % CRT+ data was recorded. A progression of the gating strategy can be seen in **Figure S9A** for both the unstained/untreated cells (top) and for cells that received 500 Hz NTP treatment (bottom). As it can be clearly seen, the unstained cell population had nearly no positive PI or CRT populations (<1%), while the NTP-treated and stained group was 81.4% PI– and 8.11% CRT+ (**Figure S9A**).

In order to determine the actual % CRT+ population, cellular auto-fluorescence and non-specific binding of the antibody must be accounted for. Therefore, each untreated or treated sample was split into 2 vials: 1 stained with the monoclonal primary CRT antibody and the other stained with the corresponding isotype (**Figure S9B**). While untreated cells had a baseline % CRT+ population (2.79%), it was still lower than that of the 500 Hz NTP treatment (8.11%). However, as seen here, after 500 Hz treatment, even the isotype control increases its % CRT+ population. Therefore, the actual % CRT+ population was determined by subtracting the % CRT+ cells of the isotype-stained samples from that of the CRT-stained samples. This difference was reported and graphed as % CRT+/PI– throughout the manuscript.

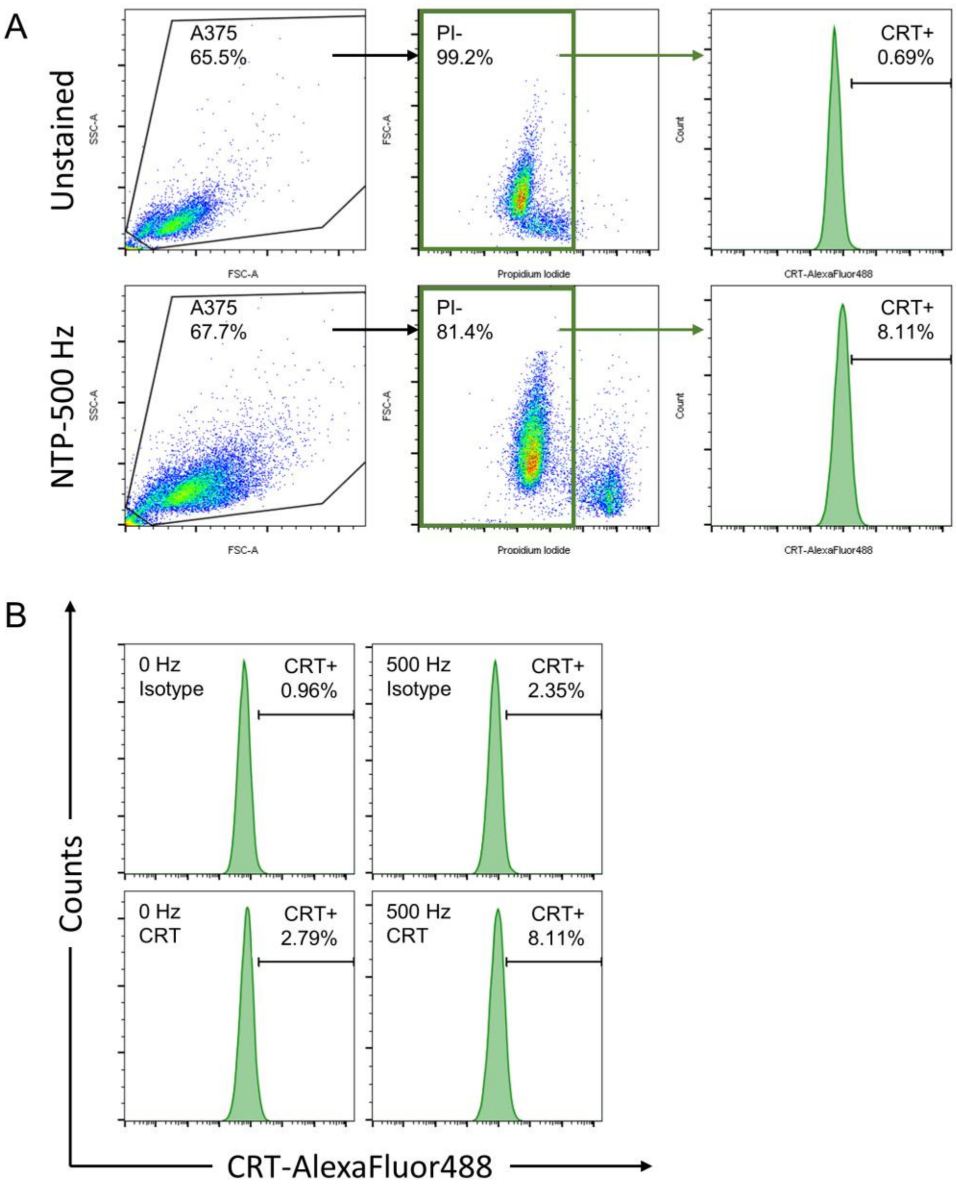

Figure S9. The gating strategy was performed in the FlowJo software and % CRT+ cells was determined based on monoclonal primary CRT staining and isotype staining. (**A**) The cell population was first gated, followed by the PI– population, and finally by the CRT+ population. (**B**) Each untreated or treated sample was split into 2 vials before staining in order to determine % CRT+ cells with isotype controls. The actual % CRT+/PI– populations are determined after subtracting each monoclonal primary CRT-stained sample from its corresponding isotype control.
